# Energy contribution from ultra processed foods in peruvian children

**DOI:** 10.17843/rpmesp.2025.423.14339

**Published:** 2025-09-29

**Authors:** Marianella Miranda-Cuadros, Miguel Campos-Sánchez, Gustavo Cediel Giraldo, María Laura da Costa Louzada, Joaquín Alejandro Marrón-Ponce

**Affiliations:** 1 Instituto Nacional de Salud, Lima, Peru. Instituto Nacional de Salud Lima Peru; 2 Universidad Peruana Cayetano Heredia, Lima, Peru. Universidad Peruana Cayetano Heredia Universidad Peruana Cayetano Heredia Lima Peru; 3 Universidad de Antioquía, Medellín, Colombia. Universidad de Antioquia Universidad de Antioquía Medellín Colombia; 4 Universidad de Sao Paulo, Sao Paulo, Brasil. Universidade de São Paulo Universidad de Sao Paulo Sao Paulo Brazil; 5 Instituto Nacional de Salud Pública, Cuernavaca, México. Instituto Nacional de Salud Pública Cuernavaca México

**Keywords:** Peru, Children, Food Intake, Energy Intake, Ultra processed Foods

## Abstract

**Objective.:**

To evaluate the energy contribution of ultra-processed foods (UPFs) and its association with social and demographic covariates in children between 6 and 35 months of age, based on national surveys conducted in 2008‒2010, 2015‒2016, and 2019.

**Materials and methods.:**

The surveys used multistage stratified random samples. 24-hour recalls were applied on random days per participant, using the modified multiple-pass method, with the support of visual aids and scales. UPFs belonged to the Nova 4 group. Given the bimodal distribution, the covariates were analyzed using two models: a binomial model for the percentage of UPF consumers and a normal model for the average energy contribution, only among UPF consumers. The estimates and models were adjusted according to the sampling design.

**Results.:**

2887 children were included. UPFs contributed 27% (95% CI: 25 to 29) of the total energy intake and were consumed by 86% (84 to 89) of children. The main energy contribution from UPFs came from the milk and dairy products group (19% [17 to 20]) and cereals (5% [4 to 6]). The covariates associated with consumption were age, calendar quarter, and poverty. No associations were found with sex or the year of the survey.

**Conclusions.:**

In children aged 6 to 35 months in Peru, UPFs provided an average of 27% of total energy and were consumed by 86%. The main sources were the milk and dairy products, and the cereals group. UPF consumption was associated with age, poverty, and the calendar quarter.

## INTRODUCTION

Overweight and obesity represent current challenges to global health ^1^. They are clear determinants of serious chronic diseases ^2^^,^^3^. Their global prevalence is increasing in both youth and adults ^4^^,^^5^, and is linked to the multiple burden of malnutrition throughout the life cycle ^6^. Although to a lesser extent, they are also present in infants and children ^7^^,^^8^. In Peru, a prevalence of 9% for both conditions has been reported in children under five years of age ^9^^,^^10^.

One of the main determinants of overweight and obesity is the consumption of ultra-processed foods (UPFs) ^11^^-^^13^. According to the World Health Organization (WHO), UPFs are “industrial formulations made wholly or mostly from substances derived from foods and additives, with little if any intact Group 1 food. Additives are used to imitate and intensify the sensory qualities of unprocessed or minimally processed products and dishes, and meals prepared with those products and with processed culinary ingredients” ^14^. UPFs include foods that, to varying degrees, contribute excess energy, sugars, sodium, trans and saturated fats to the diet ^15^, as well as non-caloric additives and sweeteners, which lack nutritional value ^16^. The consumption of UPFs has been associated with adiposity in older children ^11^^,^^12^ and with cardiovascular and metabolic diseases in adults ^17^^-^^20^.

There is an increasing number of studies on the consumption of UPFs in young children. A multinational review ^21^ reported that the intake of UPFs in children aged 2 to 5 years represented 44% of the total energy intake in Chile, 38% in Mexico, 27% in Argentina, and 18% in Colombia ^22^^-^^25^. In Peru, no previous estimates of UPF consumption in young children have been published.

This study aims to estimate the energy contribution of UPFs and its association with some social and demographic covariates in children aged 6 to 35 months in Peru, using two national surveys: National Monitoring of Nutritional Indicators (MONIN) ^26^ and Food and Nutritional Surveillance by Life Stages (VIANEV) ^27^^-^^29^. These results will be relevant to support strategies and policies aimed at promoting healthy eating and controlling overweight and obesity.

**KEY MESSAGES**

Motivation for the study. Obesity is a growing global problem in children. Ultra-processed foods (UPFs) are an important determinant, and their consumption level among children in Peru has not yet been reported.

Main findings. The results indicate that in children aged 6 to 35 months, UPFs contribute 27% of the energy consumed, are ingested by 86% of children, come mainly from dairy products and cereals, and their consumption varies according to age, poverty, and seasonality.

Implications for public health. We propose indicators to monitor UPF consumption, evaluate whether interventions reduce it, and identify its determinants. We recommend examining the benefits and risks associated with the consumption of evaporated milk in children.

## MATERIALS AND METHODS

This article presents a secondary analysis of two repeated cross-sectional national surveys, with multistage stratified random sampling, which evaluated children’s diet through a 24-hour recall (24hR): MONIN (2008-2010) and VIANEV (2015-2016 and 2019).

### Population and sample

MONIN included children under five years of age, dividing the country into five geographical areas: Metropolitan Lima (provinces of Lima and Callao), rest of the coast (western Andes below 2000 m a.s.l.), urban highlands (populated centers at 2000 m or more a.s.l. with 2000 or more inhabitants), rural highlands (populated centers at 2000 m or more a.s.l. with less than 2000 inhabitants), and jungle (eastern Andes below 2000 m a.s.l.); between November 2007 and April 2010.

VIANEV included children under three years of age, dividing the country into three geographical areas: Metropolitan Lima, urban, and rural, in three collection periods: October-December 2015, April-July 2016, and August-October 2019.

The universe included all residents of Peru in the indicated ages and periods. The sampling design was stratified and two-stage. The strata corresponded to the geographical domains, subdivided according to the census economic level. In the first stage, the sampling frame was the list of census clusters defined by the National Institute of Statistics and Informatics (INEI). In each stratum, clusters were selected by random sampling without replacement, with probability proportional to size (approximately 100 households per cluster), and were randomly assigned to the scheduled weeks. In the second stage, the field teams enumerated the eligible households in the selected clusters and took a simple random sample of households without replacement (10 in MONIN and 5 in VIANEV). The original sample weights were calculated as the inverse of the product of the selection probabilities in both stages. These probabilities were rescaled to the projected population for each year (supplementary material section S3).

For this article, the inclusion criteria were: children between 6 and 35 months of age, at least one 24hR with recorded energy, height-for-age z-score, and identification of the district of residence. From a total of 3022 children in that age range with a 24hR in the studied periods, 2887 were included.

### Measurement of food consumption

To evaluate the diet, a complete list of foods and beverages consumed during the 24 hours preceding the interview was recorded, using the modified multiple-pass methodology ^30^ and excluding any forgotten foods. The informant was the person in charge of preparing and serving the food, generally being the child’s mother. In cases where the child was a beneficiary of Cuna Más, a social program of daycare centers for daytime care ^31^, the program managers were interviewed.

The 24hR was applied on a random day. After the random selection of clusters in each period, they were reordered using a pre-established random permutation in a weekly sequence. Subsequently, the 10 planned households per week were also reordered using a random permutation of seven days (with three additional days selected by simple random sampling). The 24hR interview was arranged with the household within three days of the assigned random day.

The collection of the 24hR was supported by visual aids (food models and picture charts), precision scales, and tables of food composition and equivalences of household measures for foods and preparations ^32^^,^^33^. Information on breastfeeding and dietary supplements was not recorded. The training of the interviewers consisted of a 12-day workshop that included two pilot sessions in the field, in both surveys.

### Classification of foods according to industrial processing

The criteria for categorizing foods followed the Nova classification (supplementary material section S1). UPFs correspond to the Nova 4 category.

Each food or beverage was classified using a computerized sequence of rules that assigned the corresponding Nova group, based on data contained in the compiled food composition table (CENAN/ANDREA group: cereals, fruits, etc.), food codes, and key terms (required as present or absent in the description). The rules were defined by an expert nutritionist in a spreadsheet format, which a programmer reviewed to verify the adequacy of the format (spacing, use of capitalization, special characters, and logical expressions).

The supplementary material (Table S102) describes the rules used for each food group. For example, in the cereals group, whole grains or seeds, flours, flakes, and noodles were classified as Nova 1; cornstarch as Nova 2; freshly baked bread and pre-cooked sweetened flakes as Nova 3; and labeled bread and cereal products of various commercial brands as Nova 4. For labeled products not included in the food composition tables, manufacturers’ websites were consulted, as well as the Open Food Facts database ^34^.

### Measurement of covariates

The following covariates were included (supplementary material section S5): age (calculated as the difference in days between the interview date and the declared date of birth, divided by 30.4375 to obtain approximate months in floating-point format, with an approximate precision of 16 decimal digits), sex, geographical area (regrouped into Metropolitan Lima, urban, and rural), time (day of the year, day of the week, quarter, and year of survey), and district poverty (proportion of households in the district of residence with income below the poverty line, according to the INEI poverty map projections for 2009, 2013, and 2018, prior to the survey year). These descriptive covariates have been previously referred to in the literature ^21^^-^^25^. Variables such as educational level and macro-region were not included due to heterogeneous coding between the surveys.

### Data analysis

The data were recorded on printed forms during the interview and later digitized into a database with anonymized files. For this article, the analytical files were consolidated by homogenizing the names and types of variables, and selecting the subjects who met the inclusion criteria.

For each subject, the total energy intake and the energy contribution of each Nova category were calculated using the compiled food composition table. This table included data obtained through direct chemical analyses continuously performed by CENAN ^32^, as well as information from external sources ^33^, and from product labels collected during the interviews.

The energy contribution of UPFs was the main outcome variable of the study, defined individually as the percentage of energy consumed (kcal/24h) that comes from UPFs (variable E4RZ), measured on a random surveyed day. Upon examining the distribution of this variable (supplementary material section S2), a bimodality was identified under exploratory logarithmic transformation, with a peak of non-consumers (E4RZ=0) and another of consumers (E4RZ>0). For this reason, a normalizing transformation (Box-Cox) was sought, which resulted close to the power of 0.5, i.e., the square root.

Consequently, three per capita indicators were used. For the descriptive results, indicator ^1^ MAAT was used, the arithmetic mean of the individual contributions E4RZ (untransformed) in the total population of children. For the analytical results, two other indicators were used: ^2^ POCT, the percentage of individuals who consumed UPFs in the total population of children, and ^3^ MRAT, the retransformed mean, calculated as the square of the arithmetic mean of the square roots of the E4RZ contributions, but only among UPF consumers. MRAT is expressed in the same percentage scale as MAAT and is calculated in a subset of the total population.

MAAT, as “energy contribution,” is the indicator usually reported in the literature ^(22-25)^. The association with the covariates was evaluated using two generalized linear models: ^1^ binomial for POCT, and ^2^ normal for MRAT. POCT and MRAT are not alternative indicators for the same outcome, but a complementary set of indicators and models that, together, express the energy contribution of UPFs. Rather than two separate models, it can be considered a single model with two components.

Processing and analysis were performed with R software version 4.4.2, using the survey, ggplot, and dplyr packages. All estimates, models, and confidence limits were adjusted according to the sampling design and weighting (supplementary material sections S3 to S6). The preprint of this article is available at: https://www.medrxiv.org/content/10.1101/2024.05.28.24308069v1. The data and analysis program can be consulted at: https://github.com/vipermcs/pdata/blob/main/UPJPEDR2023.zip. A supplement with technical details and expanded results is attached to this publication.

## RESULTS

Table 1 presents the weighted distribution of the social and demographic covariates corresponding to the 2887 children included from both surveys. It is observed that age and sex are distributed uniformly. By design, MONIN oversamples the urban area and the fourth quarter of the year, while VIANEV oversamples the rural area and the second quarter, and undersamples the first. All results presented are weighted, adjusting for these sampling differences.

**Table 1:** Distribution of covariate levels, overall and by survey

Covariate/category		Total, n=2887	MONIN, n=723	VIANEV, n=2164
Area				
	Metropolitan Lima	29.2 (24.2 to 34.2)	30.9 (22.8 to 38.9)	26.6 (23.8 to 29.5)
	Urban	53.1 (48.4 to 57.8)	51.3 (45.1 to 57.4)	55.8 (48.5 to 63.1)
	Rural	17.8 (15.2 to 20.3)	17.9 (14.1 to 21.6)	17.6 (14.7 to 20.5)
Sex				
	Female	49.2 (44.6 to 53.8)	49.8 (42.9 to 56.6)	48.3 (43.2 to 53.3)
	Male	50.8 (46.3 to 55.3)	50.2 (43.5 to 57.0)	51.7 (46.8 to 56.7)
Age				
	6-11 months	21.5 (18.0 to 25.1)	22.7 (17 to 28.4)	19.8 (17.2 to 22.3)
	12-17 months	18.7 (16.1 to 21.2)	18.2 (14.3 to 22.1)	19.4 (17.1 to 21.7)
	18-23 months	19.3 (16.8 to 21.8)	18.3 (14.5 to 22.1)	20.7 (18.2 a 23.2)
	24-29 months	19.5 (16.8 to 22.2)	19.5 (15.4 to 23.5)	19.7 (16.7 to 22.6)
	30-35 months	21.0 (18.2 to 23.8)	21.4 (17.1 to 25.7)	20.5 (17.4 to 23.5)
Poverty				
	0-14%	24.4 (19.9 to 29)	18.4 (12.2 to 24.5)	33.6 (27.1 to 40.2)
	15-19%	14.8 (9.9 to 19.7)	13.3 (5.8 to 20.7)	17.2 (12.2 to 22.2)
	20-39%	35.4 (28.6 to 42.1)	38.9 (28.7 to 49.2)	29.9 (23.1 to 36.7)
	40-90%	25.4 (20.9 to 29.9)	29.4 (23.3 to 35.6)	19.3 (12.9 to 25.7)
Quarter				
	January-March	17.7 (13 to 22.5)	25.3 (18.1 to 32.4)	6.3 (1.1 to 11.5)
	April-June	28.2 (21.6 to 34.9)	28.2 (17.5 to 38.9)	28.2 (24 to 32.5)
	July-September	22.8 (19.5 to 26.1)	17.1 (12.2 to 22.0)	31.4 (27.5 to 35.3)
	October-December	31.3 (25.2 to 37.3)	29.4 (20.8 to 37.9)	34.1 (26.3 to 41.9)

Percentage estimates (95% confidence intervals), adjusted for sample design and weighting.

MONIN: National Monitoring of Nutritional Indicators. VIANEV: Food and Nutritional Surveillance by Life Stages.

**Figure 1:**
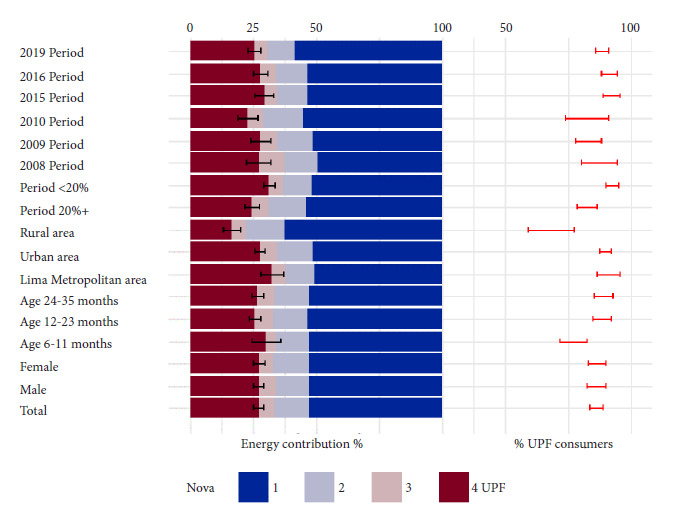
(Unweighted) distribution of the sample by surveys and covariates

Figure 1 shows the energy contribution of each Nova category (left side, Nova 4 corresponds to the MAAT indicator) and the percentage of UPF consumers (right side, corresponds to the POCT indicator), both in the total population and according to covariate categories. Table 2 presents the detailed estimates of these quantities. The national MAAT indicator was 27% (95% CI: 25 to 29). The national POCT indicator was 86% (95% CI: 84 to 89). Some possible differences are observed according to age, poverty, and geographical area, which are analyzed below using adjusted models.

Table 3 presents the MAAT indicator broken down by Nova category and food group. Among UPFs, the milk and dairy products group was the main contributor with 18.6% (95% CI: 17.2 to 20.0), followed by the cereals and derivatives group with 4.5% (95% CI: 3.6 to 5.5) (supplementary material section S7).

Figure 1. has two parts. The left panel presents the energy contributions according to processing for the total population and for the population in each category of each covariate. The right panel presents the proportions of children who consume ultra-processed foods (UPF) for the total and each category of each covariate. In the left panel, stacked bars represent on the horizontal axis, with different colors, the energy contribution of each Nova category (1: unprocessed or minimally processed foods, 2: processed culinary ingredients, 3: processed foods, and 4: ultra-processed foods, UPF), which is the MAAT indicator (arithmetic mean of individual [untransformed] contributions in the total pediatric population). For UPF, bars covering the 95% confidence intervals are also presented. In the right panel, red error bars represent on the horizontal axis the 95% confidence intervals of the POCT indicator (percentage of individuals who consumed UPF in the total pediatric population). The horizontal scales for MAAT and POCT are at the top. The total sample was 2,887 children.

**Table 2:** Processing indicators by covariate level, totals and by survey.

Survey	Covariates	N	Contribution Nova 1 (%)	Contribution Nova 2 (%)	Contribution Nova 3 (%)	Contribution Nova 4 (UPF) MAAT (%)	UPF Consumers POCT (%)
Total		2887	53 (52 to 55)	13 (13 to 14)	6 (6 to 7)	27 (25 to 29)	86 (84 to 89)
	Male sex	1457	53 (52 to 55)	13 (12 to 14)	7 (6 to 8)	27 (25 to 29)	86 (83 to 90)
	Female sex	1430	53 (51 to 55)	14 (13 to 15)	6 (5 to 7)	27 (25 to 29)	87 (83 to 90)
	Age 6-11 months	591	53 (48 to 58)	13 (11 to 15)	4 (3 to 5)	30 (24 to 36)	77 (72 to 83)
	Age 12-23 months	1168	54 (52 to 56)	14 (12 to 15)	7 (6 to 8)	25 (23 to 27)	89 (85 to 92)
	Age 24-35 months	1128	53 (51 to 55)	14 (12 to 15)	7 (6 to 8)	27 (25 to 29)	89 (85 to 93)
	Area Metropolitan Lima	749	51 (47 to 54)	11 (10 to 13)	6 (5 to 7)	32 (28 to 37)	91 (87 to 95)
	Urban area	1176	52 (50 to 53)	14 (13 to 15)	7 (6 to 8)	27 (26 to 29)	90 (88 to 92)
	Rural area	962	63 (59 to 66)	16 (13 to 18)	5 (4 to 7)	16 (13 to 20)	68 (59 to 77)
	Period 2008	68	49 (45 to 54)	13 (10 to 16)	11 (6 to 16)	27 (22 to 32)	87 (80 to 95)
	Period 2009	483	51 (48 to 55)	14 (12 to 16)	7 (6 to 8)	28 (24 to 32)	83 (78 to 88)
	Period 2010	172	55 (51 to 59)	16 (13 to 18)	6 (5 to 8)	23 (19 to 27)	83 (74 to 91)
	Period 2015	558	53 (51 to 56)	12 (11 to 13)	6 (4 to 7)	29 (26 to 33)	92 (89 to 95)
	Period 2016	643	53 (51 to 56)	13 (12 to 14)	6 (5 a 7)	28 (25 to 30)	91 (88 to 94)
	Period 2019	963	59 (56 to 61)	11 (10 to 12)	5 (5 to 6)	25 (23 to 28)	88 (86 to 91)
	Poverty 20%+	1700	54 (52 to 56)	15 (13 to 16)	7 (6 to 8)	24 (21 to 27)	82 (79 to 86)
	Poverty <20%	1187	52 (50 to 54)	11 (11 to 12)	6 (5 to 6)	31 (29 to 33)	92 (90 to 95)
MONIN		723	52 (50 to 54)	14 (13 to 16)	7 (6 to 8)	27 (24 to 29)	83 (80 to 87)
	Male sex	352	52 (49 to 55)	14 (12 to 15)	8 (7 to 9)	27 (23 to 30)	83 (78 to 89)
	Female sex	371	52 (49 to 55)	15 (13 to 17)	6 (5 to 7)	27 (23 to 30)	84 (79 to 89)
	Age 6-11 months	152	50 (43 to 57)	14 (11 to 17)	4 (3 to 6)	31 (23 to 40)	76 (68 to 84)
	Age 12-23 months	281	52 (48 to 55)	15 (13 to 17)	8 (7 to 10)	25 (22 a 28)	86 (80 to 92)
	Age 24-35 months	290	53 (50 to 56)	14 (12 to 16)	7 (6 to 8)	25 (22 to 28)	86 (80 to 92)
	Area Metropolitan Lima	141	49 (44 to 54)	12 (9 to 14)	6 (5 to 8)	33 (26 to 40)	89 (83 to 96)
	Urban area	438	50 (48 to 52)	15 (13 to 17)	8 (6 to 10)	27 (24 to 29)	88 (84 to 92)
	Rural area	144	63 (57 to 68)	17 (14 to 20)	5 (3 to 7)	15 (10 to 20)	60 (46 to 75)
	Period 2008	68	49 (45 to 54)	13 (10 to 16)	11 (6 to 16)	27 (22 to 32)	87 (80 to 95)
	Period 2009	483	51 (48 to 55)	14 (12 a 16)	7 (6 to 8)	28 (24 to 32)	83 (78 to 88)
	Period 2010	172	55 (51 to 59)	16 (13 to 18)	6 (5 to 8)	23 (19 to 27)	83 (74 to 91)
	Poverty 20%+	525	53 (50 to 56)	16 (14 to 18)	7 (6 to 9)	24 (20 to 28)	80 (75 to 86)
	Poverty <20%	198	51 (48 to 54)	12 (10 to 13)	6 (5 to 7)	31 (28 to 35)	90 (86 to 95)
VIANEV		2164	55 (54 to 56)	12 (11 to 12)	6 (5 to 6)	27 (26 to 29)	91 (89 to 93)
	Male sex	1105	55 (53 to 57)	12 (11 to 13)	6 (5 to 6)	27 (25 to 29)	90 (88 to 93)
	Female sex	1059	55 (53 to 57)	12 (11 to 13)	6 (5 to 7)	28 (25 to 30)	91 (88 to 94)
	Age 06-11 months	439	58 (54 to 62)	11 (9 to 13)	4 (2 to 5)	28 (24 to 32)	79 (74 to 84)
	Age 12-23 months	887	57 (55 to 59)	12 (11 to 13)	6 (5 to 6)	26 (24 to 28)	93 (90 to 95)
	Age 24-35 months	838	52 (50 to 54)	12 (11 to 13)	7 (6 to 7)	29 (27 to 31)	95 (93 to 97)
	Area Metropolitan Lima	608	53 (51 to 55)	11 (9 to12)	5 (4 to 6)	31 (28 to 33)	94 (92 to 96)
	Urban area	738	53 (52 to 55)	12 (11 to 13)	6 (5 to 7)	29 (26 to 32)	93 (90 to 95)
	Rural area	818	63 (60 to 66)	13 (12 to 15)	6 (5 to 7)	18 (16 to 21)	79 (75 to 84)
	Period 2015	558	53 (51 to 56)	12 (11 to 13)	6 (4 to 7)	29 (26 to 33)	92 (89 to 95)
	Period 2016	643	53 (51 to 56)	13 (12 to 14)	6 (5 to 7)	28 (25 to 30)	91 (88 to 94)
	Period 2019	963	59 (56 to 61)	11 (10 to 12)	5 (5 to 6)	25 (23 to 28)	88 (86 to 91)
	Poverty 20%+	1175	57 (55 to 59)	13 (12 to 14)	6 (5 to 7)	24 (22 to 26)	87 (84 to 90)
	Poverty <20%	989	53 (51 to 55)	11 (10 to 12)	5 (4 to 6)	31 (28 to 33)	94 (92 to 96)

Estimates (95% confidence intervals) adjusted for sample design and weighting. Only Nova 3 Age 6-11m and period 2008 had a coefficient of variation >15 (15.4 and 24.5, respectively).

UPF: ultra-processed foods (Nova 4). MAAT: arithmetic mean of individual contributions (untransformed) in the total population. POCT: percentage of individuals who consumed UPF in the total population. MONIN: National Monitoring of Nutritional Indicators. VIANEV: Food and Nutritional Surveillance by Life Stages.

**Table 3:** Average energy contribution of each food group by processing level, totals and by survey.

Food group		Total %	MONIN %	VIANEV %
Unprocessed or minimally processed				
	Cereals and derivatives	20.7 (19.6 to 21.8)	20.4 (18.5 to 22.2)	21.1 (20.2 to 22.0)
	Vegetables and derivatives	0.9 (0.8 to 1.0)	0.9 (0.7 to 1.0)	0.9 (0.8 to 1.0)
	Fruits and derivatives	8.6 (7.9 to 9.2)	8.2 (7.2 to 9.2)	9.1 (8.4 to 9.8)
	Fish and seafood	0.9 (0.7 to 1.1)	0.6 (0.4 to 0.9)	1.2 (0.9 to 1.6)
	Meats and derivatives	4.9 (4.4 to 5.3)	4.5 (3.8 to 5.2)	5.3 (4.9 to 5.7)
	Milk and dairy products	1.9 (1.4 to 2.4)	2.6 (1.8 to 3.5)	1.0 (0.6 to 1.3)
	Eggs and derivatives	3.2 (2.9 to 3.4)	2.8 (2.4 to 3.2)	3.7 (3.4 to 3.9)
	Legumes and derivatives	1.8 (1.5 to 2.2)	1.9 (1.3 to 2.5)	1.8 (1.5 to 2.1)
	Tubers, roots and derivatives	5.6 (5.1 to 6.1)	4.7 (4 to 5.5)	6.7 (6 to 7.3)
	Others	2.3 (1.7 to 2.8)	3.2 (2.3 to 4.2)	1.1 (0.7 to 1.4)
Ingredients				
	Fats, oils and oilseeds	6.2 (5.0 to 7.4)	8 (5.8 to 10.1)	3.9 (3.4 to 4.4)
	Sugary products	7.5 (6.8 to 8.2)	7.1 (6 to 8.3)	8.0 (7.3 to 8.7)
	Others	0.3 (0.2 to 0.4)	0.2 (0.1 to 0.4)	0.4 (0.2 to 0.7)
Processed				
	Cereals and derivatives	4.9 (4.5 to 5.3)	5.1 (4.5 to 5.7)	4.6 (4.1 to 5.1)
	Milk and dairy products	0.6 (0.5 to 0.7)	0.5 (0.3 to 0.7)	0.7 (0.6 to 0.9)
	Not a simple food	0.8 (0.3 to 1.3)	1.4 (0.6 to 2.3)	0.0 (0.0 to 0.0)
	Others	0.2 (0.1 to 0.3)	0.2 (0.1 to 0.2)	0.3 (0.1 to 0.4)
Ultra-processed (UPF)				
	Cereals and derivatives	4.5 (3.6 to 5.5)	4.7 (3.0 to 6.3)	4.3 (3.9 to 4.8)
	Milk and dairy products	18.6 (17.2 to 20)	17.7 (15.4 to 20.0)	19.8 (18.3 to 20.0)
	Sugary products	1.1 (0.7 to 1.4)	1.2 (0.6 to 1.8)	0.9 (0.7 to 1.2)
	Others	4.6 (4.0 to 5.1)	4.1 (3.1 to 5.0)	5.2 (4.6 to 5.8)

MAAT estimates (95% confidence intervals) adjusted for sample design and weighting.

MAAT: arithmetic mean of individual contributions (untransformed) in the total population. MONIN: National Monitoring of Nutritional Indicators. VIANEV: Food and Nutritional Surveillance by Life Stages

**Figure 2:**
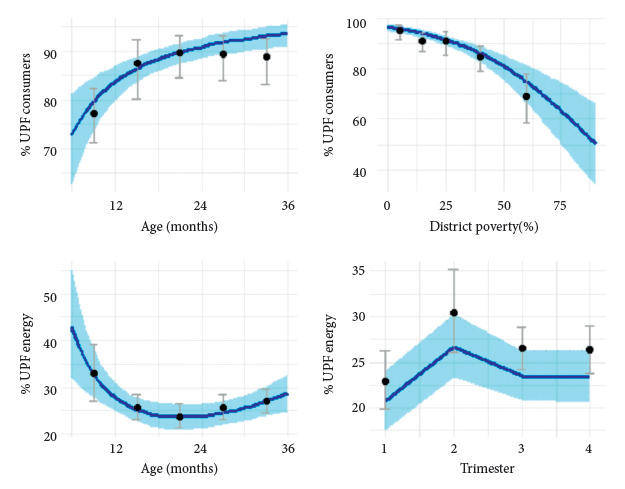
Modeling of indicators by covariates.

Table 4 and Figure 2 present the results of the adjusted models. In the binomial model, the POCT indicator clearly increases with age and decreases with poverty. In the normal model, the MRAT indicator decreases with age, although it shows a slight increase in the older age groups, and is somewhat lower in the austral summer and higher in autumn. The variations by age do not follow a linear trend. The modeling did not identify significant associations with other covariates (sex, geographical area, survey year, day of the year, or month), nor with interaction terms. The review of model assumptions and diagnostics (supplementary material section S6) indicates that the residual distributions are satisfactory, with some significances attributable to type I error.

**Table 4:** Models of ultra-processed food consumption indicators by covariate levels, totals and by survey.

Survey	Model	Term	Coefficient	Standard Error	p-value
Total	Binomial	Intercept	0.377	0.650	0.562
		Age, logarithm	0.943	0.207	0.000
		District poverty, %	-0.036	0.005	0.000
	Normal	Intercept	10.917	1.876	0.000
		Age, m	0.145	0.046	0.002
		Age, logarithm	-3.088	0.945	0.001
		April-June	0.614	0.244	0.012
		July-September	0.296	0.204	0.148
MONIN	Binomial	October-December	0.284	0.214	0.186
		Intercept	0.680	0.876	0.438
		Age, logarithm	0.800	0.276	0.004
		District poverty, %	-0.037	0.007	0.000
	Normal	Intercept	11.248	2.527	0.000
		Age, m	0.133	0.064	0.039
		Age, logarithm	-3.124	1.282	0.016
		April-June	0.878	0.322	0.007
		July-September	0.452	0.284	0.113
		October-December	0.218	0.250	0.384
VIANEV	Binomial	Intercept	-0.615	0.561	0.273
		Age, logarithm	1.316	0.195	0.000
		District poverty, %	-0.029	0.006	0.000
	Normal	Intercept	9.655	1.578	0.000
		Age, m	0.142	0.040	0.001
		Age, logarithm	-2.612	0.806	0.001
		April-June	0.154	0.434	0.723
		July-September	0.113	0.431	0.794
		October-December	0.289	0.483	0.551

Estimates adjusted for sample design and weighting. The weighting of both surveys has been homologated by rescaling it to the projected population for each year.

Normal model of MRAT: retransformed mean (square of the arithmetic mean of the square roots of individual contributions only in the population of ultra-processed food consumers). Binomial model of POCT: percentage of individuals who consumed ultra-processed foods in the total population. MONIN: National Monitoring of Nutritional Indicators. VIANEV: Food and Nutritional Surveillance by Life Stages.

Figure 2 presents the models adjusted for covariates in four panels. The binomial model (n=2,887) is in the two lower panels. The normal model (n=2,531) is in the two upper panels. Each panel presents the scenario for one of the covariates in each model. The vertical axis presents the dependent variable. For the binomial model, the dependent variable is the POCT indicator (percentage of individuals who consumed ultra-processed foods [UPF] in the total pediatric population). For the normal model, the dependent variable is the MRAT indicator (retransformed mean, the square of the arithmetic mean of the square roots of individual contributions only in the population of children consuming UPF). The horizontal axis presents the covariate. The solid blue lines present the prediction for an "average" child (21 months of age, in July-September, 30% district poverty, except for the covariate of each panel). The light blue shades are the 95% confidence bands. The black circles are the weighted estimates from the observed sample (with its actual mix of covariates), and the gray bars are their weighted 95% confidence intervals. These final models did not select interaction terms.

## DISCUSSION

This study constitutes the first estimate of UPF consumption in children in Peru. Consumption was described by two indicators: MAAT, the average energy contribution of UPFs was 27% (95% CI: 25-29), and POCT, the percentage of UPF consumers was 86% (95% CI: 84-89). Likewise, the analysis that incorporated a third indicator (MRAT, the average energy contribution only among consumers) identified age (POCT increases with age, while MRAT decreases), poverty (MRAT is lower with greater poverty), and calendar quarter (MRAT is lower in austral summer and higher in autumn) as associated covariates.

When comparing the energy contribution of UPFs in Peru (27% in children between 6 and 35 months), with that of the closest available age group for other countries (children aged 2 to 5 years) ^21^, it is observed that the Peruvian value is lower than that reported in Mexico (38%), Chile (44%), Australia (47%), the United States (58%), and the United Kingdom (61%), but higher than that recorded in Colombia (18%). Taking into account the modeling, and despite possible minor methodological differences, it is postulated that when it is calculated in the future for children aged 2 to 5 years, the comparable figure for Peru will be somewhat lower, but in the same position among countries.

It was observed that most of the UPF consumption comes from foods belonging to two groups: milk and dairy products, and cereals. Evaporated milk is the most frequent UPF, consumed by 58% (95% CI: 55 to 61) of children, and contributed 13% (95% CI: 11 to 14) of the total energy, approximately half of the energy attributed to UPFs. In contrast, fresh milk was consumed by only 7% (95% CI: 6 to 9). Evaporated milk contains sodium, saturated fats, and additives ^32^^,^^33^, and promotes the consumption of added sugars when sweetened at home (children who consumed it ingested a geometric mean of 10 g (95% CI: 9 to 12) of sugar, compared to 4 g (95% CI: 4 to 5) consumed by those who did not). On the other hand, it provides several macro and micronutrients, constitutes a source of fortification, and does not require refrigeration as long as the can remains closed. Evaporated milk has represented an important industry in Peru since approximately 1940 ^35^. The balance between these considerations for and against is a topic that requires further research, in order to generate evidence that allows for the review of recommendations on its use in children’s diets.

A limited set of covariates was chosen, corresponding to those used in previous studies ^22^^-^^25^, with the exception of the macro-regional domain, whose categories are specific to each country, and mother’s education, which in Peru is very homogeneous.

Regarding age, in other Latin American countries, the energy contribution from children over 2 years of age, as measured by MAAT, decreases with age ^22^^,^^24^^,^^25^. The present study showed something more complex: MRAT, the average energy contribution of UPFs only in consumers, decreased with age until approximately 21 months and, from that point, seemed to increase slightly, while POCT, the percentage of UPF consumers, increased with age. It is interpreted that in MAAT, the opposite component of POCT (i.e., the subjects who did not consume UPFs) is masked. A possible explanation would be the introduction of infant foods, such as those from the milk and dairy products group (particularly evaporated milk) and cereals. The energy contribution of these foods would subsequently decrease when the household diet is introduced. The sample used does not allow for precise modeling of the third year of life, and the literature also lacks data for the first two years.

Regarding the socioeconomic level, some evidence has also been reported ^22^^,^^25^ of differences similar to those observed in the present analysis, which found that MRAT decreases with the level of poverty. Likewise, there is evidence of a variable degree of association ^36^, although most studies are in adults who participated in the National Health and Nutrition Examination Survey (NHANES) ^37^^-^^39^. In a context where UPF sales are increasing ^14^, understanding the mechanisms that relate UPFs, poverty, and prices is important for designing effective interventions ^40^.

Regarding the quarterly differences, although plausible, no reports were found in the revised literature. Seasonality may be related to the availability of UPFs, climate, income, and/or expenses; however, these hypotheses have not been previously documented and warrant investigation in future studies. No differences were identified according to sex. Similarly, no differences were found by year, which could be explained if the level of poverty, which has markedly reduced in Peru during the observed period ^41^, were the primary mediator of the changes over time.

The findings presented have relevant implications for public and nutritional health. First, although there are no official international limits to qualify the health importance of UPF consumption levels, given that they are not essential foods and considering the mentioned risks, it is reasonable to keep these levels as low as possible. Second, if there were individual UPFs with benefits, they would have to be discussed and justified; in that sense, evaporated milk is a priority for study, as previously discussed. Third, the set of three indicators used in this study, in addition to others such as critical nutrients ^16^, could be incorporated into surveillance instruments, as they provide non-redundant information and contribute to refining the description and analysis. Fourth, Peruvian food labeling regulations ^42^ require octagonal warning symbols on packaging, an intervention that must continue and be reinforced, also considering the child population, and whose monitoring and evaluation requires surveillance of UPF trends in the coming years. Fifth, although the covariates identified here are descriptive, they can serve as a basis for designing studies aimed at targeting groups or intervention mechanisms.

Among the limitations of this study are the following: (1) representativeness of rural areas (although formally included with the appropriate weighting, there is an impression that field difficulties may have generated poorly documented losses); (2) recall loss (being a long procedure that requires appointments for random days, it is common in recall surveys ^43^^,^^44^ that appointments are not made or carried out; the availability of replacements preserves the target sample size, but does not necessarily compensate for possible biases; as a rough estimate, of the eligible children in the consolidated file, 96% have been included, of the apparently eligible children in the source files, 70% have been selected in the consolidated file); (3) reproducibility of the Nova classification, especially for UPFs (as has been reported in some previous works ^45^^-^^47^) and is also the experience of the team, there may be some ambiguity in the assignment of the UPF category, particularly when the description of foods may include versions with and without UPFs. While it is considered that these cases are not numerous nor do they cover the majority of the consumed mass, this eventuality can be ruled out); (4) the selection of covariates, whose intention has been basically descriptive (among other variables that could be of interest, but have not been included here, we can mention the characteristics of maternal education, the ecological region, and the prices and elasticities of foods); (5) the measurement of the poverty covariate, which is a district projection, not an individual classification, which could attenuate its effect; and (6) the measurement of consumption, which is with a single recall, does not estimate usual consumption, for which the observed variability is somewhat greater. While some of these limitations may have led to overestimations and others to underestimations, they do not weaken the study’s conclusions. However, all these aspects deserve to be addressed in future research.

Among the strengths of our study are: (1) coverage of all regional diversity; (2) large sample size, as reflected in the confidence intervals; (3) application of a standardized 24hR; (4) application of the 24hR on random days; and (5) application of statistical adjustments for bimodality (zero-inflation), asymmetry, confounding, and complex sample. These results can serve as a baseline for analyzing UPF intake in children in Peru prior to the implementation of front-of-package labeling.

It is concluded that, among children aged 6 to 35 months in Peru between 2008 and 2019, UPFs (Nova 4): (1) contribute 27% of the total energy; (2) were consumed by 86% of children, a percentage that increased with age and decreased with poverty; (3) the UPFs with the greatest energy contribution are those from the milk and dairy products, and cereals groups; and (4) among consumers, the energy contribution decreased with age and showed seasonal variation. It is recommended that, given the growing evidence against UPFs, the temporal trend should also be monitored in this age group. The desirability of discouraging the consumption of evaporated milk and cereals in this group should be reviewed. Finally, a more in-depth analysis of the consumption pattern of UPFs in children aged 6 to 59 months is pertinent, a period characterized by a particularly important nutritional dynamic.
