# Supplementary material for: Energy contribution from ultra processed foods in peruvian children
Source: Rev Peru Med Exp Salud Publica. 2025 Sep 29;42(3):240–51. doi: 10.17843/rpmesp.2025.423.14339 (PMC12679980; doi:10.17843/rpmesp.2025.423.14339)
Supplement: Supplementary material. — Available in the electronic version of the RPMESP. [file rpmesp-42-03-14339-s001.pdf]

Suplemento

Versión 2025-May-12.

Suplemento para:

Contribución energética de los alimentos ultraprocesados en niños peruanos: frecuencia, bimodalidad y covariables asociadas.

Por:

Marianella Miranda-Cuadros, mymirandac@gmail.com

Miguel Campos-Sánchez

Gustavo Cediel

María Laura da Costa Louzada

Joaquín Alejandro Marrón-Ponce

El presente suplemento incluye, parcialmente traducido, el contenido del suplemento de la prepublicación https://www.medrxiv.org/content/10.1101/2024.05.24.24307617v1 , con las salidas actualizadas de los modelos y la adición de tablas suplementarias y fragmentos actualizados del programa. Para los resultados descriptivos incluidos aquí se mantienen las mismas variables de la prepublicación. Los resultados del modelamiento se han modificado ligeramente, porque la publicación ya no incluyó las variables antropométricas.

Contenido

Sección S1 Clasificación NOVA 3
Tabla S101 Definiciones de la Clasificación Nova 3
Tabla S102 Categorías Nova para Alimentos y Bebidas 7
Sección S2 Distribución de la Variable Resultado (Outcome) 11
Figura G002 Ingesta de Alimentos Ultraprocesados (Ponderada) 11
Figura S002 Transformaciones (No Ponderadas de la Ingesta de Alimentos Ultraprocesados 12
Sección S3 Técnicas de Procesamiento de Datos 13
Archivos de Entrada 13
Flujo de Trabajo 15
Paquetes de Software R 16
Disponibilidad de Datos 16
Sección S4 Modelamiento Estadístico de Covariables 17
Sección S5 Distribuciones y Asociaciones de Covariables 19
Figura G101 Distribución de la Muestra a lo largo del Tiempo (Ponderada) 19
Figura G201 Distribuciones de y Asociaciones entre Covariables (No ponderadas) 20
Sección S6 Diagnósticos de los Modelos 21
Figura S004 Diagnósticos de Modelos con Residuos Cuantílicos (Expandidos) 22
Sección S7 Fuentes de Alimentos Ultraprocesados 23
Figura G223 Distribución de Grupos de Alimentos que proporcionan Nova 4 (Ponderada) .. 23
Sección S8 Tablas Suplementarias 24
Tabla S801 Contribución Energética Promedio, Calorías 24
Tabla S811 Distribución de la Población según Encuestas y Covariables 25
Tabla S812 Indicadores de Consumo según Encuesta, Proceso y Covariables 26
Tabla S813 Contribución Energética Promedio según Encuesta, Proceso y Grupo 27
Tabla S814 Modelamiento de Indicadores según Encuesta y Covariables 28
Sección S9 Fragmentos Actualizados del Programa 29
Sección SA Referencias Adicionales 31

Sección S1 Clasificación NOVA

Citando la reciente guía FAO (Monteiro et al 2019a):

> The NOVA classification system groups all foods according to the nature, extent and purposes of the industrial processes they undergo. These involve physical, biological and chemical techniques used after foods are separated from nature, and before they are consumed or else made into dishes and meals.

La siguiente Tabla S101 es una adaptación de la Tabla 1 de la version castellana de dicha publicación, incorporando aclaraciones de otras fuentes (Monteiro et al 2019a, Monteiro et al 2019b, FAO 2021, PAHO 2019). A partir de esta guía se prepare una secuencia de reglas programadas, como se menciona en el artículo principal. La Tabla S102 presenta un resumen verbal de dicha secuencia de reglas. Ambas tables. S101 and S102 se presentan aquí en castellano, como fueron originalmente usadas.

Tabla S101 Definiciones de la Clasificación Nova

Grupo 1: Alimentos no o mínimamente procesados.
Los alimentos sin procesar son partes comestibles de plantas (semillas, frutos, hojas, tallos, raíces) o animales (músculos, despojos, huevos, leche), y también de hongos y algas, así como el agua, después de separarlos de la naturaleza.<br>Los alimentos mínimamente procesados son alimentos alterados por procesos como la remoción de partes no comestibles o no deseadas, o bien como el secado, trituración, molienda, desmenuzamiento, filtración, tostado, ebullición, pasteurización, refrigeración, congelación, colocación en recipientes, envasado al vacío o fermentación no alcohólica. Su objetivo principal es prolongar la vida de los granos (cereales), legumbres (legumbres), verduras, frutas, nueces, leche, carne y otros alimentos, permitiendo su almacenamiento para un uso más prolongado y, a menudo, para que su preparación sea más fácil o diversa. En ninguno de estos procesos se agregan sustancias como sal, azúcar, aceites o grasas o edulcorantes a los alimentos originales.<br>Alimentos con vitaminas y minerales agregados, en general para reemplazar los nutrientes perdidos durante el procesamiento, como harina de trigo o maíz fortificados con hierro o ácido fólico. Los elementos del grupo 1 no suelen contener aditivos para preservar sus propiedades originales (1) o pueden con poca frecuencia contener aditivos <u>que prolongan la duración del producto, protegen propiedades originales o prevenir la proliferación de microorganismos.</u><br>Incluye hierbas y especias utilizadas en preparaciones culinarias, tales como tomillo, orégano, menta, pimienta, clavo y canela, entera o en polvo, fresca o seca.

Grupo 2: Ingredientes culinarios procesados.
Se trata de sustancias obtenidas directamente de los alimentos del grupo 1 o de la naturaleza por medio de procesos como el prensado, refinado, trituración, molienda, extracción o minería y secado por atomización. Normalmente no se consumen por sí solos, sino que se usan principalmente como ingredientes para preparar, sazonar y cocinar los alimentos del grupo 1 y para que los platos, sopas, panes, ensaladas, bebidas, postres y otras preparaciones culinarias hechas a mano tengan un sabor agradable y sean variados, nutritivos y placenteros.<br>Los ejemplos son la sal gema o marina, el azúcar y la melaza obtenidos a partir de la caña o la remolacha, la miel extraída de los panales y el jarabe obtenido del arce y los aceites vegetales prensados de las aceitunas o semillas, y la grasa obtenidas a partir de la leche (mantequilla) y de la carne de cerdo (manteca), y los almidones extraídos del maíz y otras plantas (féculas/maicena). Los productos que constan de dos elementos del grupo 2, como la mantequilla salada, los elementos del grupo 2 con vitaminas o minerales agregados, como la sal yodada, y el vinagre obtenido mediante fermentación acética del vino u otras bebidas alcohólicas permanecen en este grupo. Los elementos del grupo 2 pueden contener aditivos para preservar las propiedades originales del producto.

Grupo 3: Alimentos procesados.
Son los productos relativamente sencillos preparados añadiendo azúcar, aceite, sal u otras sustancias del grupo 2 a los alimentos del grupo 1. La mayoría de los alimentos procesados contienen dos o tres ingredientes. Los procesos a los que se someten incluyen diversos métodos de preservación o cocción y, en el caso de los panes y el queso, la fermentación no alcohólica.<br>La finalidad principal de la elaboración de productos procesados es aumentar la durabilidad de los alimentos del grupo 1 o modificar o mejorar sus cualidades sensoriales.<br>Algunos ejemplos son las verduras, frutas y legumbres enlatadas o embotelladas conservadas en almíbar o salmuera, los frutos secos y semillas endulzados o salados, las carnes saladas, curadas o ahumadas (jamón, tocino, pastrami), el pescado enlatado en aceite, sal y/o ahumado, la fruta en forma de jarabe, y la mayoría de panes recién horneados y quesos artesanales a los que se agrega sal.<br>Los productos alimenticios procesados generalmente conservan la identidad básica y la mayoría de los constituyentes del producto original.<br>Los alimentos procesados pueden contener aditivos para preservar sus propiedades originales o prevenir la contaminación microbiana. Algunos ejemplos son la fruta en forma de jarabe con antioxidantes agregados y las carnes secas saladas con agentes conservantes agregados.

Grupo 4: Alimentos ultraprocesados.
Son formulaciones industriales que suelen estar compuestas por cinco o más ingredientes. Además de la sal, azúcar, aceites y grasas, entre los ingredientes de los alimentos ultraprocesados se incluyen sustancias alimentarias que generalmente no se emplean en las preparaciones culinarias, como variedades de azúcares (fructosa, jarabe de maíz con alto contenido de fructosa, "concentrados de jugo de frutas", azúcar invertido, maltodextrina, dextrosa , lactosa), aceites modificados (hidrogenados o aceites interesterificados) y fuentes de proteínas (proteínas hidrolizadas, aislado de proteína de soja, gluten, caseína, proteínas de suero y “carne separada mecánicamente”) y los aditivos usados para imitar las cualidades sensoriales de los alimentos sin procesar o mínimamente procesados y sus preparaciones culinarias o para esconder cualidades indeseables del producto final, como **colorantes, aromatizantes**, **potenciadores del sabor**, edulcorantes sin azúcar (aspartamo, el ciclamato o los compuestos derivados de la stevia) , emulsificantes (espesantes), sales **emulsionantes**, humectantes, secuestrantes, reafirmantes, aumentadores de volumen, antiespumantes, antiaglomerantes, gelificantes, agentes de recubrimiento y de glaseado. A menudo se expresan como una clase, como saborizantes o sabores naturales o sabores artificiales; o sus nombres van seguidos de su clase, como 'glutamato monosódico (potenciador del sabor)', o 'color caramelo', o 'lecitina de soja como emulsionante. En cualquier caso, el Codex Alimentarius de la ONU proporciona una lista actualizada periódicamente de aditivos con sus clases funcionales así como un servicio de búsqueda en línea donde se pueden buscar nombres y clases de aditivos.<br>La finalidad principal del ultraprocesado industrial es crear productos listos para comer, beber o calentar, susceptibles de sustituir los alimentos sin procesar o mínimamente procesados, así como los platos recién preparados.<br>Algunos ejemplos de alimentos ultraprocesados son las bebidas con gas (carbonatados) ; los snacks dulces o salados envasados; los helados, el chocolate y los caramelos (dulces); los panes y bollos envasados producidos en masa; la margarina y otros productos para untar; el queso procesado; las galletas (bizcochos), pasteles, tortas y mezclas para pasteles; los cereales para el desayuno, las barritas de cereales y energéticas; las bebidas energéticas; las bebidas lácteas; el yogur y bebidas de fruta; las bebidas de cacao; los extractos y las salsas instantáneas de carne y pollo; las fórmulas para lactantes, las leches de continuación y otros productos para lactantes; los productos “sanos” y de “adelgazamiento” como los sustitutos de comida “enriquecidos” o en polvo; y muchos productos listos para calentar, como los pasteles y la pasta y la pizza previamente preparados; los trocitos y barritas de pollo y pescados; las salchichas, hamburguesas y perritos calientes, y otros productos cárnicos reconstituidos y las sopas, fideos y postres instantáneos envasados y en polvo. Las formulaciones y los ingredientes utilizados en su pre elaboración los hacen ultraprocesados.<br>Los productos elaborados exclusivamente con alimentos del grupo 1 o el grupo 3 que también contienen aditivos “cosméticos” o potenciadores sensoriales, como el yogur natural con edulcorantes artificiales y los panes con emulsionantes, se incluyen aquí en el grupo 4.

Tabla S102 Categorías Nova para Alimentos y Bebidas

	Grupo de Alimentos	Descripción	Grupo Nova
A	Cereales y derivados		
		granos o semillas enteros o partidas o pelados : quinua, trigo, cebada, maíz, arroz, etc	1
		granos o semillas presentados en harinas, sémola, polenta	1
		granos o semillas presentados como Hojuelas	1
		fideos (excepto instantáneos)	1
		hojuela de cereales fortificada y precocida	1
		maicena/maizena	2
		hojuela de cereales fortificada y precocida+ azúcar	3
		"Pan" o similares y pasteles (sin marca) recién horneados	3
		"Pan" o similares y pasteles con marcas	4
		productos a base de cereales: cerevita, kiwigen	4
		pan de "molde"	4
		keke de"tienda"	4
		Galletas	4
		Panetón	4
		Tostadas	4
		barras energéticas con cereales	4
		Barquillos	4
		Hojuela de cereales fortificada y precocida+ azúcar +aditivos	4
		corn flakes	4
		cereales infantiles envasados (nestum, cerelac,etc)	4
B	Vegetales y derivados		
		verduras al natural y en condición cocida	1
		encurtidos o en salmuera (con conservantes)	3
		salsa de tomate (sin aditivo o con conservante como ácido cítrico)	3
		salsa de tomate concentrada, con o sin carne (con otros aditivos) , ketchup	4
		encurtidos o en salmuera (con conservantes y otros tipos de aditivos)	4
C	Frutas y derivados		
		frutas al natural y en condición cocida	1
		agua o jugo natural de fruta (100%) o pasteurizado, sin azúcar	1
		jugo de frutas, pulpa	1
		jugo de frutas endulzada (con preservante como ácido cítrico)	3
		néctar de pulpa de fruta, endulzada	3
		néctar (contiene aditivo conservante como ácido citrico)	3
		néctar (contiene aditivo como ácido cítrico y otros , stevia u otro edulcorante)	4
		frutas en almibar, frutas confitadas	4

D	Grasas, aceites y oleaginosos		
		maní y otras oleaginoas	1
		cocoa	1
		grasa de animal	1
		manteca / mantequilla	2
		aceites/oil	2
		mantequilla de maní	3
		mantequilla de maní (con aditivos)	4
		Chocolate	4
		productos como milo, nescao o similares	4
		Margarina	4
		maní confitado	4
E	Pescados y mariscos		
		Pescados o mariscos al natural o en condición cocida	1
		sardina, atún u otros, enlatados/en conserva, en aceite y/o sal y/o salsa de tomate	3
		pescados o mariscos secos y/o salados	3
		pescado o mariscos deshidratados o curados	3
F	Carnes y derivados		
		Carne de cualquier animal, cruda o en condición cocida	1
		carne semi seca, seca, ahumada, oreada y/o salada	3
		charqui/chalona/charque	3
		tocino, cecina	3
		carne o derivados en conserva (en sal y/o aceite)	3
		hamburguesas, salchichas, embutidos, jamón	4
		carnes empanados tipo “nuggets”	4
G	Lácteos y derivados		
		Leche de "animal", leche "entera", leche "natural", leche fresca	1
		cuajada, suero de leche	1
		leche "pasteurizada" o "ultrapasteurizada" líquida	1
		yogurt natural (leche y cultivos caso la marca "Danlac")	1
		quesos (con sal)	3
		Quesillo	3
		yogures y bebidas lácteas aromatizados y endulzados, leche cultivada	4
		leche "saborizada"	4
		crema de leche	4
		leche condensada	4
		queso fundido	4
		leche "evaporada" (entera, descremada, semidescremada, light, deslactosada, UHT)	4
		queso crema, fundido	4
		yogur natural (con aditivo como pectina)	4
		chocolatada envasada (Gloria, Laive…)	4
		Manjarblanco	4
		leche en polvo (con aditivo no conservante caso Gloria, Pura Vida, etc)	4
		mezcla o producto lácteo, sucedáneos	4

H	Bebidas Alcohólicas y Analcohólicas		
		sin azúcar	1
		cerveza	3
		vino	3
		néctar (pulpa, jugo, agua y azúcar)	3
		néctar (contiene aditivo: ácido cítrico)	3
		néctar (contiene aditivo como ácido cítrico, otros, stevia)	4
		ron, coñac, similares	4
		kola (cola) , gaseosas	4
		chocolatada envasada (Gloria, Laive, etc)	4
		suero oral	4
		bebida de soya envasada	4
J	Huevos y derivados		
		huevos frescos y en diferentes estados de cocción	1
		deshidratados, desecados	1
K	Azucarados		
		caña de azúcar	1
		miel, chancaca	2
		Azúcar	2
		jaleas, jarabe	2
		Mermeladas	4
		Gelatina	4
		algodón de azúcar	4
		azúcar impalpable	4
L	Miscelánea		
		Achiote, algas, azafrán, yuyo, cocoa, comino, algarrobo en polvo, champiñones, laurel, pimienta, anís, clavo, canela, nuez, seta, hongo, kion, cushuro, cebada en polvo	1
		te (hojas), café grano o molido,	1
		Sal	2
		Levadura	2
		vinagre (no sintético)	2
		vinagre sintético (etanol)	4
		sillao /salsa de soja	4
		"en almíbar"	4
		caldo de res/pollo/gallina	4
		kectchup, gelatina	4
		Mayonesa	4
		salsas envasadas	4
		café en polvo	4
		cereal bar	4
		Helado	4

Q	Infantiles		
		papillas de programas sociales	1
		papilla o colado (Heinz y similar)	3
		sucedáneos de leche materna	4
		cereales en lata (Nestum, Cerelac, etc)	4
T	Leguminosas y derivados		
		todos los frijoles o leguminosas, incluidos sus harinas	1
		frijol remojado enlatado (sin aditivo)	3
		bebidas o "leches" de menestras, envasadas	4
U	Tubérculos, raíces y derivados		
		En estado natural o cocido	1
		harinas o en polvo	1
		Deshidratado	1
		puré deshidratado	4
		hojuela para puré	4
V	Tubérculos Andinos		
		En estado natural o cocido	1

Sección S2 Distribución de la Variable Resultado (Outcome)

Initially, the outcome variable was the energy intake fraction provided by UPF, that is, the total energy provided by UPF divided by the total energy intake, both per 24h.

Figura G002 Ingesta de Alimentos Ultraprocesados (Ponderada)

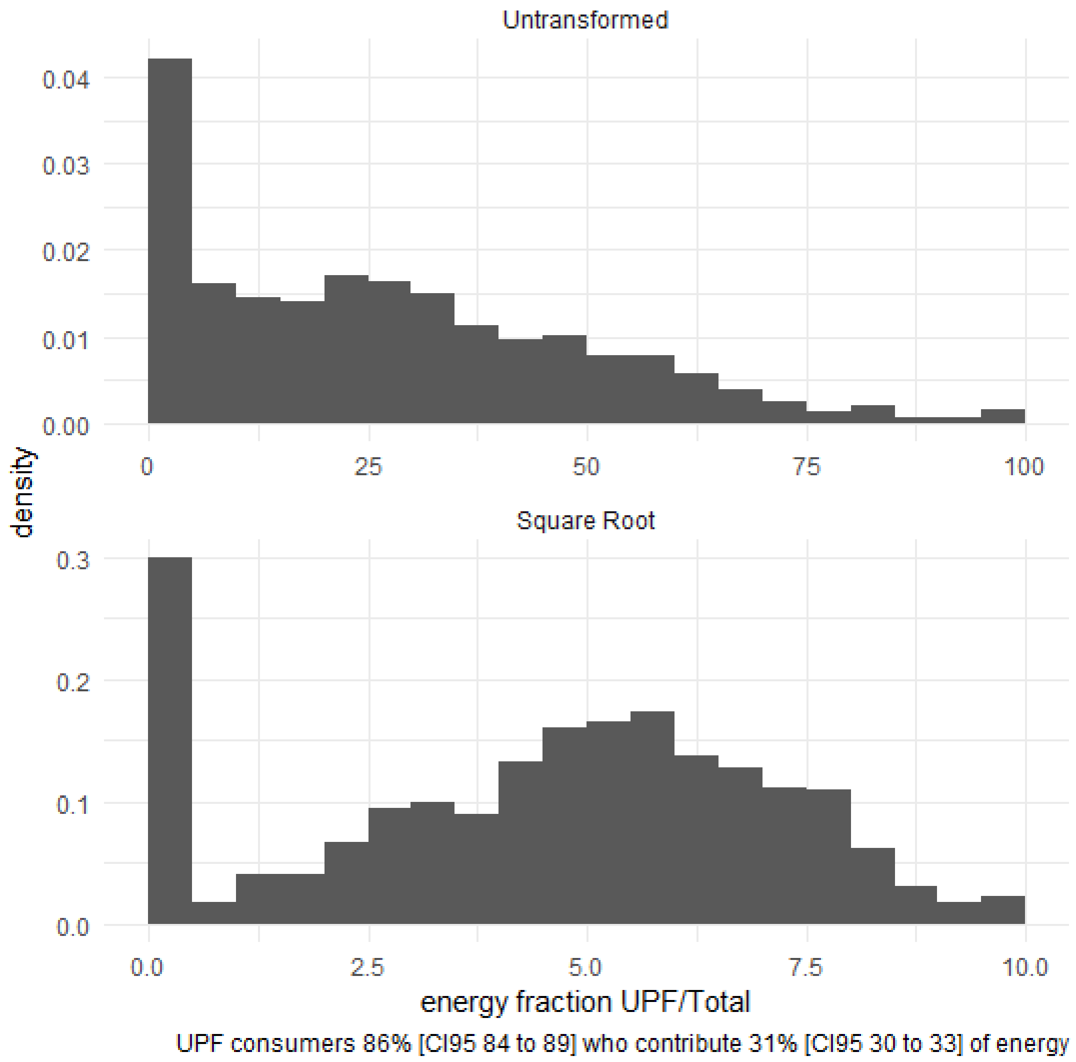

The upper panel shows the frequency distribution of the untransformed energy fraction among the 2887 children, adjusted for the complex sampling design. The horizontal axis has the energy fraction, expressed as percentage. The vertical axis has the frequency, expressed as the probability density. The lower panel shows the frequency distribution of the square root of that energy fraction, adjusted for the complex sampling design.

The transformed variable is clearly bimodal. The left peak has 14% [CI95 11 to 16] children who were not UPF consumers. The right peak has the remaining 86% [CI95 84 to 89] children who were UPF consumers.

Figura S002 Transformaciones (No Ponderadas de la Ingesta de Alimentos Ultraprocesados

To arrive at the square root transformation, Box-Cox (in four variants) and Tukey transformations were fitted to the unweighted data for consumers only, obtaining the following lambda and gamma coefficients for the best fit in each transformation.

Method	Package	Function	Option	Lambda	Gam	Min	Max
Box & Cox	car	powerTransform	bcPower	0.490	NA	-2.02	0
Yeo & Johnson	car	powerTransform	yjPower	-1.35	NA	0.0000495	0.451
Hawkins & Weisberg	car	powerTransform	bcnPower	0.262	0.1	-2.08	0.00249
Tukey	rcompanion	transformTukey	<NA>	0.625	NA	0.00204	1
Box & Cox	MASS	boxcox	<NA>	0.45	NA	-2.20	0

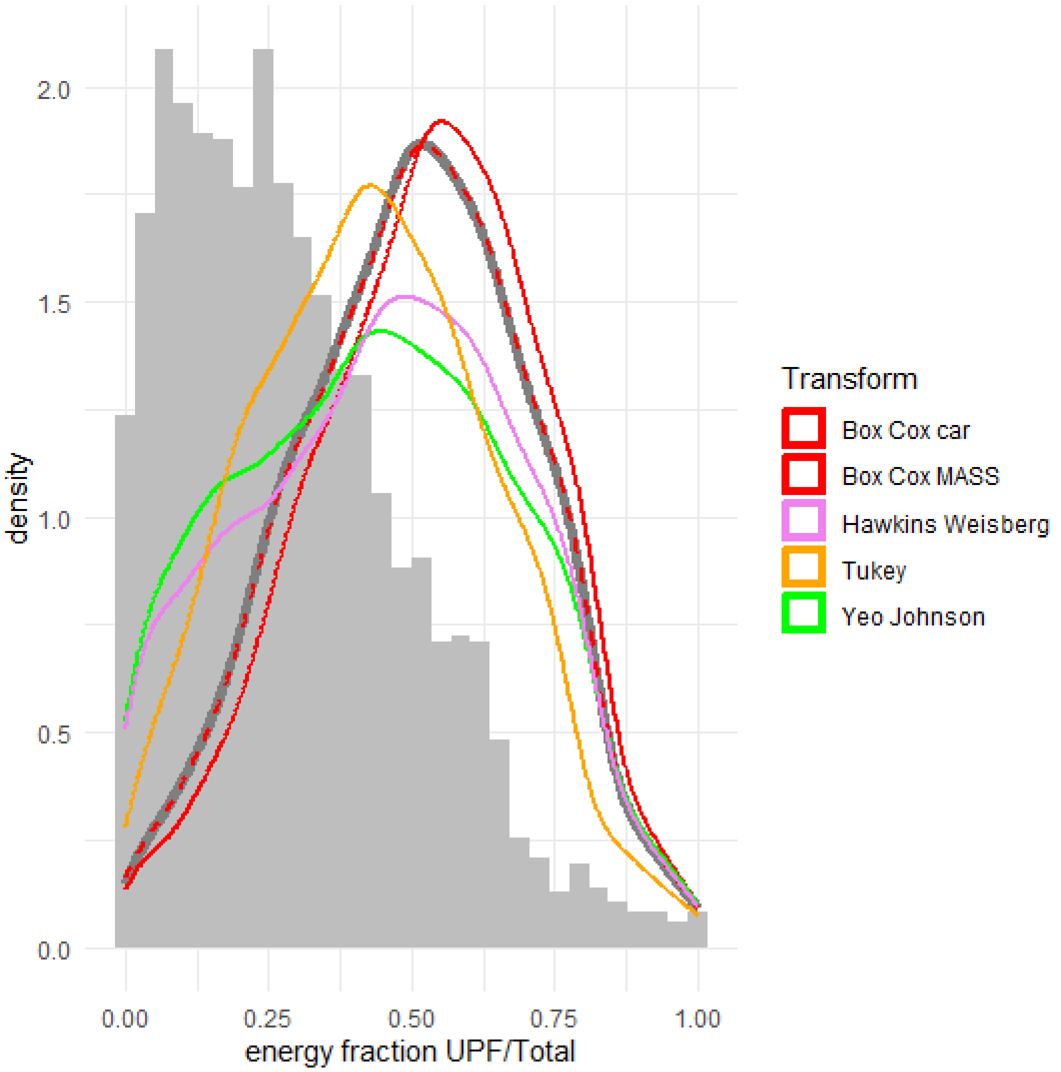

The density distributions of the original and transformed (rescaled to the range [0,1]) variables are shown. The square root transformation has been added, and finally chosen, because the Box -Cox lambda estimates are close to 0.5 and it is a simple transform.

Sección S3 Técnicas de Procesamiento de Datos

Archivos de Entrada

The input files for the processing are listed below, together with their MD5 signatures:

```
   PART  FNAM                                                   BYT MD5                              FEC
   <chr> <chr>                                                <dbl> <chr>                            <chr>
 1 LIBS  NuTabs.rda                                          594646 66404cdac1771bb9d3bda6a42cf10393 2023-02-01 10:18:26
 2 LIBS  GCTabs.rda                                          320674 dad57e6b3fbb948d0a318a51a2e6d1d9 2021-08-13 13:46:11
 3 LIBS  PETabs.rda                                          722325 a707649f46cf49ace28ed05ee4983774 2023-08-13 22:39:52
 4 LIBS  TUBIGC.csv                                          211997 163f17d6c7b786580b490008b3d085e0 2023-09-08 12:17:44
 5 VN15  BD.01.Caratula-Caract.Viv.sav                       648123 c91e092330d885e5658623b815e50c59 2018-01-30 08:34:52
 6 VN15  BD.03.Salud.y.Nutricion.sav                         160411 e2e843c8cea13643b05890c31fe9ca88 2018-01-30 08:37:01
 7 VN15  BD.02.Caract-Miemb.Hogar.sav                        278996 9ac40fc1bcf471d2b22393d507d9aab2 2018-01-30 08:36:18
 8 VN15  BD.09.Consumo.Ind-Niño-Hogar.sav                   7500562 a15568088efc62522999cdd983493480 2018-01-30 08:42:38
 9 VN15  BD.05.Consumo.Ind-Niño-Cuna.Mas.sav                  90981 7f5058cff8319565dd8562f0575f22c8 2018-01-30 08:39:31
10 VN15  BD.10.Preparacion.Ind-Niño-Hogar.sav               6596076 3a5003a1b93f6656828587e3b44efc6f 2018-01-30 08:43:02
11 VN15  BD.06.Prepar-Cuna.Mas.sav                           187642 ed622a5375cd7330c6b25f29e5b13234 2018-01-30 08:42:04
12 VN16  CAP001.sav                                          788455 54c33b08eb37fa1a7023143629daf773 2017-03-16 22:39:24
13 VN16  CAP200.sav                                          567101 c589c1e4a626a87730f249f195cb2e2c 2017-03-16 22:49:59
14 VN16  CAP400.sav                                          321462 f27074330d0eb1eccc1dcc1530b622bf 2017-04-13 15:44:30
15 VN16  CAP618.sav                                         4284755 7466a6285952517cd363129cf582dd02 2017-04-13 22:30:23
16 VN16  CAP600.sav                                           34149 c936dfca59bcfdad83bdf02540dfc49a 2017-03-16 23:06:50
17 VN16  CAP623.sav                                         4548241 a4795d9125bab3e5d5483a0419f9535d 2017-04-13 23:25:05
18 VN16  CAP606.sav                                          100013 e39ed65539793efd3bf933945d368856 2017-03-17 14:38:32
19 VN19  NIÑOS 2019 INDI CONSU HAB.sav                      1613211 cb1cde5f20815af218f13198f1f645d2 2023-09-19 20:56:17
20 VN19  CAP 621 CONSUM INDIV HOGAR1.xlsx                   5252152 775a797e198ae00161d07683f316a270 2023-09-19 20:56:09
21 VN19  CAP 600 CONSUM INDIV CUNA MAS.xlsx                   42779 2ee4bfb3b3fd13f1ffbabf1eed9b6ff9 2023-09-19 20:56:09
22 VN19  CAP 627 PREP ELAB HOGAR_ CONSISTENCIA MINIMA.xlsx 11568742 0d6a8f2710399e30eafe38b2cbeda354 2023-09-19 20:56:09
23 VN19  CAP 607 PREP ELAB CUNA MAS.xlsx                     141326 de88ad61fb6e2e2c373fb57cacc6ef1b 2023-09-19 20:56:09
24 VN19  L2022123019v.rds                                      5148 6abc489064b0d65ec960c006a0cd3050 2023-09-19 20:59:17
25 MON3  V12.MDB                                           31039488 667937e87ebe8a8aee0e3c8376875334 2012-12-06 12:33:24
26 MON3  WT.sav                                             2066651 441c24dd757bcd0e7cd8d827635dff68 2014-04-13 21:45:56
27 MON3  P10.sav                                            1710283 b4385287c6c29fc8080e0f9efbbc86f8 2010-12-21 14:20:12
28 REFS  03 TABLA DE ALIMENTOS2016.xlsx                      649086 7a672c95aa1f21670ecb56b97a8ffda5 2021-04-30 13:35:56
29 REFS  TABLA DE ALIMENTOS.sav                              469462 2a6aa4dd8dbc20d3d8437e6216a25cc0 2021-07-06 18:34:54
30 REFS  TABLA ALIM INDUSTR.sav                              258736 cacd57ff68d093a2eb2ca1ad8e83bb5d 2021-07-06 18:36:38
31 REFS  L2023020109_sintaxis en excel1lg.xlsx                33776 9672c464db5bd364791c9006a667bd60 2023-03-19 09:34:10
32 REFS  L2023112217_NOVA  preparaciones -221123.xlsx         56051 05194b31a8510e71b32a0162b820cecc 2023-11-23 17:24:31
```

The PART=LIBS files are publicly available sets of several reference tables and R functions built by us for several data analysis tasks, apart from this article (Campos 2021).

The NUTABS file contains consolidated food composition tables for Peru (INS/CENAN 2017, PRISMA 2003), and household measure equivalence tables (Miranda et al 1996) as well as collected food equivalence tables used by the MONIN III survey (INS/CENAN 2023).

The NUTABS file also contains reference tables and functions for computing the recommended dietary energy requirements (FAO/WHO 2004 and IOM 2005).

The GCTABS file contains consolidated anthropometry tables and functions for the WHO Reference Data (WHO 2006).

The PETABS file contains several consolidated tables for Peru, from which we have used in this article the official population projections (INEI 2009) and the poverty maps (INEI 2010, 2015 2020).

The PART=VN15 files are consolidated data for the VIANEV 2015 survey (INS/CENAN 2023). The PART=VN16 files are consolidated data for the VIANEV 2016 survey (INS/CENAN 2023). The PART=VN19 files are consolidated data for the VIANEV 2019 survey (INS/CENAN 2023). L2022123019v is a patch file containing the district code for some households with missing data (but cluster identification number).

The PART=MON3 files are consolidated data for the MONIN survey which had food intake recalls between 2008 and 2010 (INS/CENAN 2023).

The PART=REFS files contain food composition data which were added to the consolidated NUTABS files from added foods in the VIANEV surveys and the rules for NOVA classification (a main rules file and a patch for initially unclassified foods, mainly drinks and prepared meals).

The MONIN and VIANEV data files for this article may have some discrepancies with the data files as officially published in the references quoted above (official repository publication of open data has only been available for INS/CENAN since the second half of 2023). Our impression is that those discrepancies are annoying but minor, and due to some disorder in the file housekeeping. Some details follow.

The VIANEV data had not been made public by the time this article was being prepared. Only some reports had been produced (INS/CENAN 2018, 2021, 2023) but data was publicly available only for 2015. In Peru such files can be obtained by any citizen through a Transparency of Information request, but they also can be obtained through internal official channels within INS/CENAN, which is the way we had access. The data we received had some differences in data structure between years, and there were some remaining inconsistencies which were removed during the Load step.

The MONIN surveys are three cycles, MONIN I 1995-2002, MONIN II 2004-2006 and MONIN III 2007-2010 (Campos et al 2011), only the last one has been used here. The reports and data were published initially in the CENAN web site under the denomination “Biblioteca Digital en Nutrición” (BDN) where it was available for several years. That site was taken down and replaced by links in the INS/CENAN/DEVAN web site, which has been also made inactive. The data has just recently been made available again in the repository (INS/2023). In each incarnation the data files have different structures because of different ways of compiling the information. For this article we have used one of the first versions, which was stored at the BDN, supplemented by a version of such file which was prepared for the MONIN report on food intake (Miranda et al 2012) and a research thesis (Miranda 2014).

Some of the input files listed here do have personal identification information which has been dropped in the ETL process.

Flujo de Trabajo

In this section we provide a succinct description of the processing steps carried out to prepare the data before analysis. Such processing had two phases:

In the first phase, the original input files were read and consolidated files were produced. The steps during this phase were:

- Reading of VIANEV source files, with renaming and retyping of columns as necessary to have a common data structure for the three VIANEV rounds. The geographical coding for the districts was assigned here, on the basis of the region, province and district names (the first three administrative divisions of the country). No recoding or filtering were deemed necessary at this step.
- Consolidation of all VIANEV children files. Extremely low values for height (below 40 cm) and weight (below 1 kg) were recoded to missing. Age and WHO 2006 anthropometric indices were calculated. Exclusion of subjects who were outside the age range or did not have sampling weight information was carried out in this step.
- Patching of food codes for VIANEV 2015 and 2016 data, as detected by a cleaning checkout and decided upon by data inspection. All the patches are coded, and therefore registered, in the program. Most of these corrections are related to the type of presentation of the food (for instance dry versus fresh) and some clear misdoings to very different foods.
- Pooling of reference composition tables. From previous studies we had a pooled table, containing mainly several editions of the CENAN tables as well as the ANDREA table. To this base the codes for new foods registered during the VIANEV surveys were added. Mostly industrialized products for which composition was recorded from the food package labels or the manufacturer web sites. Food codes were reassigned in some cases where they overlap. The NOVA classification was applied here from the rules file.
- Consolidation of VIANEV food intake data into three intermediate files, for preparations, ingredients, and items (served or consumed). The weight as recorded in the VIANEV files has been used, without checking for household measure equivalences. Items with zero weight or lacking subject or R24 identification data were excluded. From the three intermediate files a consolidated file has been prepared containing both the items directly consumed (for instance a fruit), the ingredients directly recorded (for instance the items in a sandwich or a hot drink) or those whose weight was calculated from the household preparations and ingredients (for instance a soup, whose recipe is recorded for the family, and a certain amount has been served to the child). For meals taken at the Cuna Más social program, the composition is available. For some meals prepared outside the household (for instance from a restaurant), a reference composition is available.
- Calculation of nutrient intake for each child, essentially joining the consolidated food intake and food composition and classification tables and grouping them by child.
- Reading of MONIN source files. Since the MONIN survey had a separate, more detailed processing, most of the consolidation described for VIANEV was already done. MONIN did use the consolidated food composition table which was expanded with the new foods recorded during the MONIN survey. So, at this step only homologation of column names and types was performed.

In the second phase, the consolidated files were used to prepare additional variables necessary for the analysis. The steps during this phase were:

- Consolidation of MONIN and VIANEV surveys.
- Computation of variables and terms for analysis and modeling.
- Joining with the district poverty maps.
- Exclusion of food with invalid weight and recordings of breast feeding intakes.
- Computation of the UPF energy intake variables (consumers and energy fraction).
- Computation of transformations of nutrient intakes divided by energy intake.
- Computation of FAO/WHO and USDA DRI energy requirements.
- Joining with yearly national population projections.
- Readjustment of sampling weights to the population projections.
- Computation of the Inclusion Condition (outcome, poverty and anthropometry).

The separation between both phases is just practical. The second phase is intended to be repeated more often during the analysis, as requirements for variables could arise.

Paquetes de Software R

The following R packages (and their pre-requisites) are specifically called by the load and analysis program:

Framework: tidyverse.

Parallel Processing: future, future.apply, furrr, parallel.

Data Management: dplyr, haven, labelled, readxl, DBI, odbc, openxlsx, readr, lubridate.

Complex Samples: survey, srvyr, svydiags.

Modeling: MASS, lme4, car, rcompanion, broom, DHARMa.

Table Production: gtsummary, flextable, gt.

Graphics Production: ggplot2, colorspace, GGally, cowplot, ggmosaic.

Disponibilidad de Datos

The management and analysis program R source code in a single file and the anonymized binary file containing data frames (LDAT) and estimates (LEST) are available under an open source license at https://github.com/vipermcs/pdata . Documentation for the frames is described at the end of the program and dictionaries are embedded (labelled) in the data frames (tibbles). The current reference files NUTABS, GCTABS and PETABS are also available under and open data license at https://github.com/vipermcs/btools .

Sección S4 Modelamiento Estadístico de Covariables

The goal of the modeling process was to identify risk markers for the ultra processed food intake (UPF, i.e. NOVA 4 foods), as measured by a single 24-h recall (R24) interview.

Initially, the outcome variable was the energy intake fraction provided by UPF, that is, the total energy provided by UPF divided by the total energy intake, both per 24h.

Since the distribution of that variable was clearly asymmetric and bimodal, it was decided to build a set of two models for two outcomes:

- A discrete dichotomous variable, for all children, 1 for those children who ate UPF and 0 for those who did not.
- A continuous variable, for UP consumer children only, with the square root of the energy fraction (as a percentage).

Thus, three indicators are described in the RPMESP paper: MAAT, the arithmetic mean of individual energy fractions, POCT, the proportion of children who consumed at least one UPF, an MRAT, the back-transformed square of the arithmetic mean of the square roots of individual energy fractions only for children who had consumed at least one UPF. MAAT is the indicator quoted in the literature. POCT and MRAT are needed for the correct analysis.

An alternative indicator for MAAT, which was used for practical reasons by our preliminary reports at INS (Miranda & Campos 2021), SLAN (Miranda & Campos 2022) and the preprint (Miranda et al 2024), is the ratio of the total energy from UPF divided by the total energy intake. This aggregated index is akin to a weighed average, with children eating more energy contributing more to the indicator than those eating less energy.

The modeling technique is the generalized linear model (GLM) which is the multivariable logistic regression for the first outcome variable and the multivariable linear regression for the second variable. Both regressions were adjusted for the complex sample design.

The candidate risk factors, independent covariates, were represented as the following terms:

- EDM: age in months, with decimals (days between the interview date and the birth date, divided by 30.4375).
- BSEX: sex, dichotomous 1 male, 2 female.
- IDDOM: geographical domain, categorical (Metropolitan Lima, Urban Rest and Rural)
- PPOB: district poverty prevalence de (INEI projections)
- DAEN: interview day of year, between 1 y 365
- AAEN: interview calendar year, between 2007 y 2019
- FTEN: interview trimester, categorical 1 to 4 (as a proxy for season)
- DSEN: interview day within the week, between 1 and 7
- DMEN: interview day within month, between 1 and 31
- SDEN: trigonometrical sin of 2.π.DAEN/365.25
- EDML: natural logarithm of EDM
- EDM:BSEX: interaction, EDML.BSEX product
- IDDOM:PPOB: interaction, IDDOM.PPOB product
- DAEN:AAEN: interaction, DAEN.AAEN product

In the preprint, anthropometry covariates (HFAZ, WFHZ, their squares and their interaction) were included, but not in the RPMESP version.

The choice of square root transformation for the energy fraction outcome was based on a univariate Box-Cox adjustment whose exponent was close to 0.5. The transformed terms, such as squares, logarithms and trigonometrical functions, represent hypothesized non-linear relationships. The interaction terms represent hypothesized heterogeneities (synergies or antagonisms). This list of candidate terms has been discussed and agreed upon by the authors.

For each of the two models the following steps were carried out:

- Selection starting from the non-interaction candidate terms.
- Selection starting from the selected terms and the interaction terms.
- Verification of the selected terms.
- Diagnostic check of the final model.

Both selection steps were repeated AIC-based backward stepwise selections from 300 re-samples (using the original complex sample design) of the original data (bootstrap-like procedure to generate training samples). Those terms which were selected in more than 50% of the replications and had p-values less than 0.15 in more than 50% pf the replications were selected for the next step.

The verification step was the model fit to the original data (considered to be the testing sample), selecting for the final model those terms, single or interaction, which were statistically significant ($p<0.05$).

The diagnostic step was the examination of Cook's D scores for outliers, the examination of the variance inflation factors, both adjusted for the complex sample design, as well as the cumulated distribution of quantile residuals generated from a reweighed sample (a simple random sample of the same size as the original data obtained from the population expanded from the original data according to the sampling weights).

The GLM regression procedure uses classical techniques (McCullagh & Nelder 1989) with the necessary adjustments for the complex sample design (Lohr 2010, Lumley 2010). The selection procedure follows the advice to avoid overfitting (Harrell 2015) using a simplified implementation of bootstrap selection (Austin & Tu 2004, Rizopoulos 2023). The diagnostic procedure uses weighed estimations of D and VIF (Li & Valliant 2011, 2015, Liao 2010, Liao & Valliant 2012) as well as quantile residuals (Dunn & Smyth 1996, Austin & Tu 2004, Hartig 2022).

The software used was R 4.4.2. For GLM the svyglm function of the survey package was used. For the stepwise selection the stepAIC function from the MASS package was used. For diagnosis the svydiags and DHARMa packages were used. The implementation of the process took advantage of parallel R execution (future package) on a Windows and AMD platform.

Sección S5 Distribuciones y Asociaciones de Covariables

Figura G101 Distribución de la Muestra a lo largo del Tiempo (Ponderada)

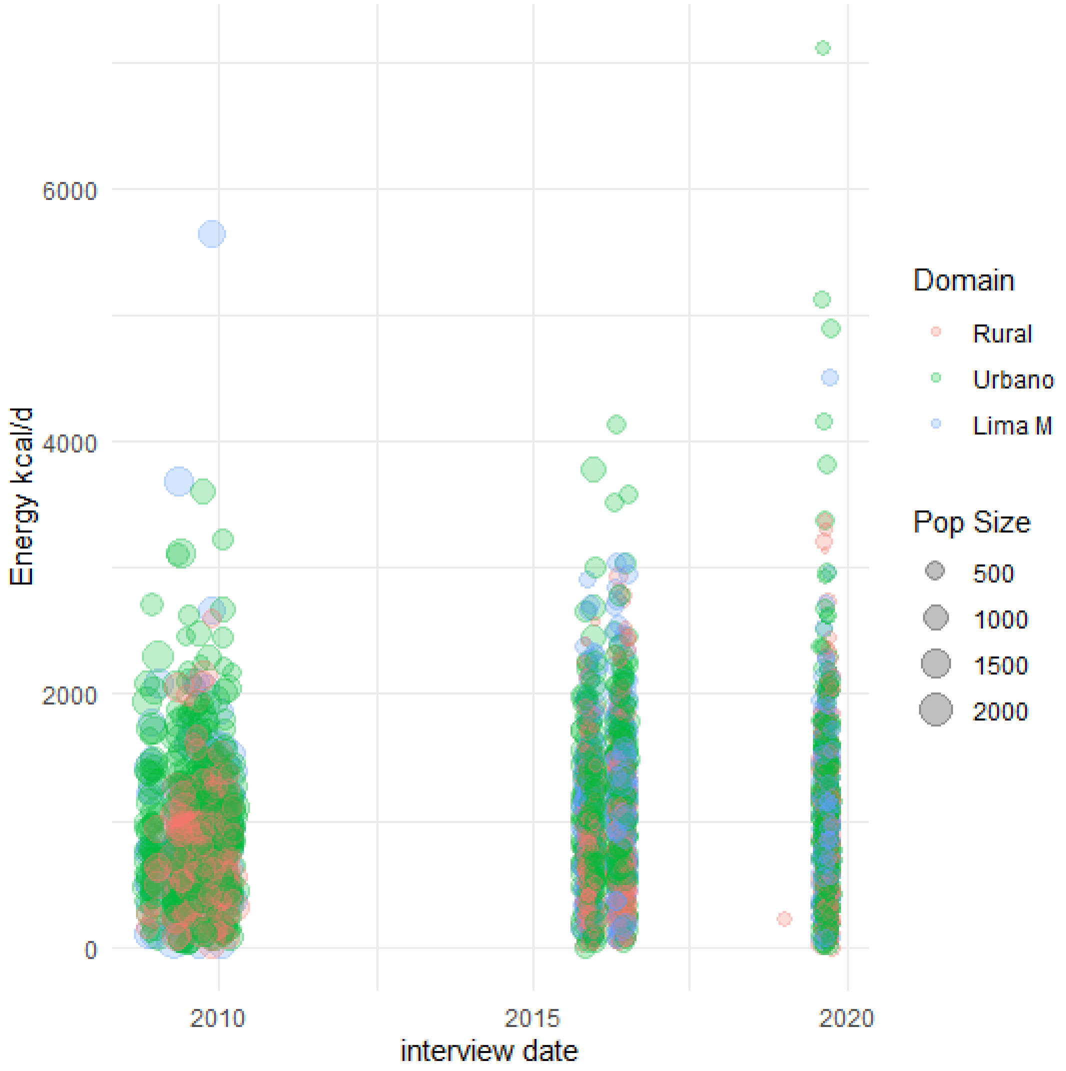

Each circle in the figure shows the energy intake value (vertical axis) and the date (horizontal value) for a sampled child. The circle diameter is proportional to the sampling weight. The colors correspond to the geographical domain. Notice that VIANEV surveys (2015, 2016 and 2018) do not cover a whole year and MONIN surveys (2008-2010) do not always cover whole years evenly.

Figura G201 Distribuciones de y Asociaciones entre Covariables (No ponderadas)

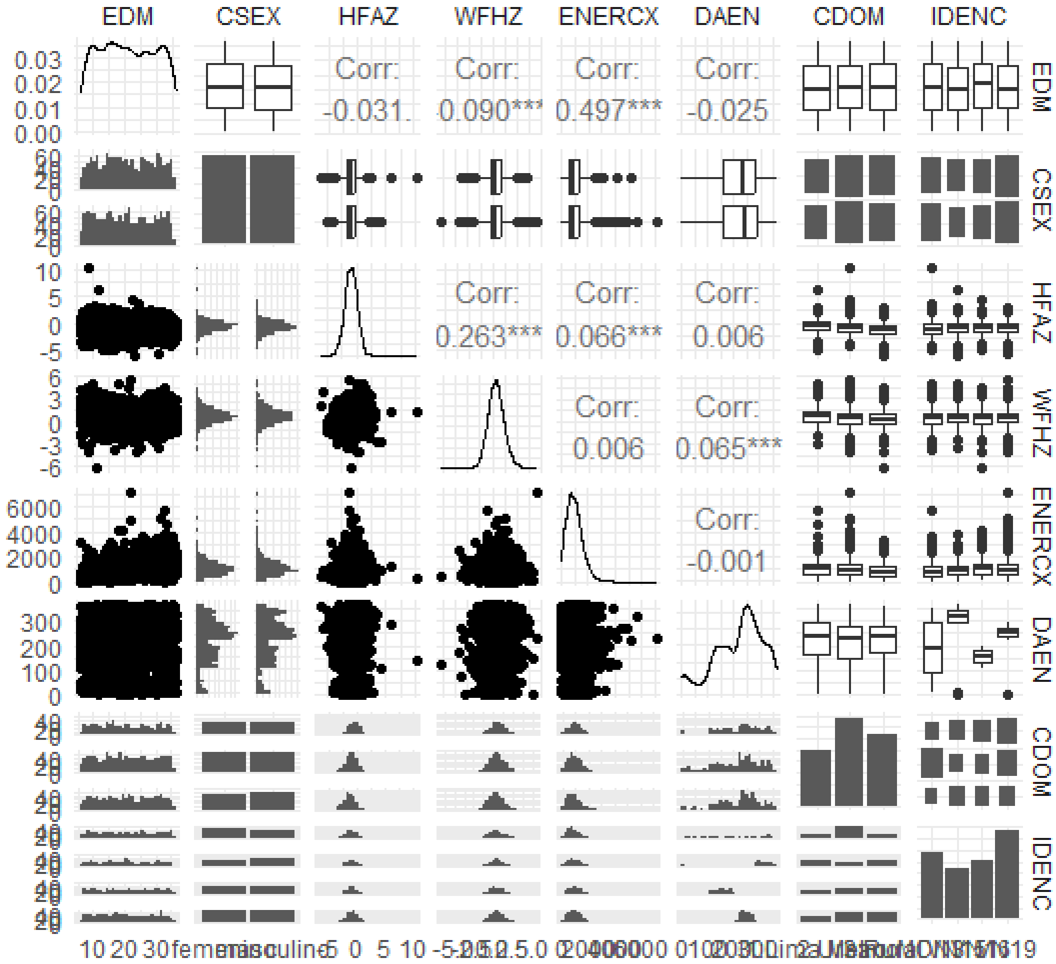

Each panel in the diagonal depicts the density distribution of a single covariate. Each panel apart from the diagonal depicts the correspondence between two covariates. Panels on the left-hand side below the diagonal show the same covariate pairs as those on the right-hand side above the diagonal, only transposed.

Sección S6 Diagnósticos de los Modelos

The transcript below shows the weighed diagnostic indices for both models (ISEQ=1 binomial and ISEQ=2 normal).

```
# A tibble: 2 × 12
   ISEQ NUOBS MCFAR2 COOKMD COOKQ1 COOKQ2  COOKP2  COOKP3 BETAPX LEVEP3 RESIP3   MCOVP0
  <int> <int>  <dbl>  <dbl>  <dbl>  <dbl>   <dbl>   <dbl>  <dbl>  <dbl>  <dbl>    <dbl>
1     1  2887 0.127  0.0553 0.0202  0.153 0.00277 0.00139 0.0187      0 0.0239 0
2     2  2531 0.0742 0.443  0.193   1.60  0.222   0.166   0.0269      0 0       0.000275
# A tibble: 13 × 11
    ISEQ TERM             TVAL  BETX    svy.vif   reg.vif       zeta        varrho zeta.x.varrho R.square FGT3
   <int> <chr>           <dbl> <int>      <dbl>     <dbl>      <dbl>         <dbl>         <dbl>    <dbl> <lgl>
 1     1 (Intercept) -85.0        0      NA        NA          NA  NA                      NA       NA     NA
 2     1 AAEN          0.0424     0 388320.    323385.      95426.  0.0000126               1.20     1.00   TRUE
 3     1 EDML          1.04      43     34.5       33.8    586862.  0.00000174              1.02     0.970  TRUE
 4     1 HFAZ          0.294     18      2.55       2.47   557887.  0.00000185              1.03     0.595  FALSE
 5     1 PPOB         -0.0305    14      4.56       4.51   465461.  0.00000217              1.01     0.778  TRUE
 6     2 (Intercept)  10.7       21     NA        NA          NA  NA                      NA       NA     NA
 7     2 EDM           0.134     31     36.5       22.3   4333882.  0.000000378             1.64     0.955  TRUE
 8     2 EDML         -2.83      28     34.2       22.4   4701988.  0.000000325             1.53     0.955  TRUE
 9     2 FTEN2         0.590     59      4.72       1.94  3550526.  0.000000685             2.43     0.485  TRUE
10     2 FTEN3         0.310     52      4.87       1.83  3144197.  0.000000848             2.67     0.453  TRUE
11     2 FTEN4         0.259     59      4.25       1.97  5012142.  0.000000429             2.15     0.493  TRUE
12     2 HFA2         -0.0264    57      0.959      1.01 11032937.  0.0000000860            0.949    0.0105 FALSE
13     2 HFAZ          0.293     22      0.929      1.02  5940090.  0.000000153             0.907    0.0237 FALSE
```

McFadden R-squared (MCFAR2) are not too high, as expected. The 50, 25 & 75 percentiles for Cook's D (COOKMD, COOKQ1, COOKQ2) are shown. The proportions of Cook's D above2 (COOKP2), above 3 (COOKP3), extreme Beta (BRTAPX), absolute Leverage over 3 (LEVEP3), residuals above 3 (RESIP3) and non-zero covariance matrix terms (MCOVP0) are shown to be very small. The variance inflation factors (svy.vif) are above 3 (FGT3) for most covariates. We interpret this as some collinearity expected (between age terms and between age and anthropometry terms) plus the noise by the complex sample design.

Figura S004 Diagnósticos de Modelos con Residuos Cuantílicos (Expandidos)

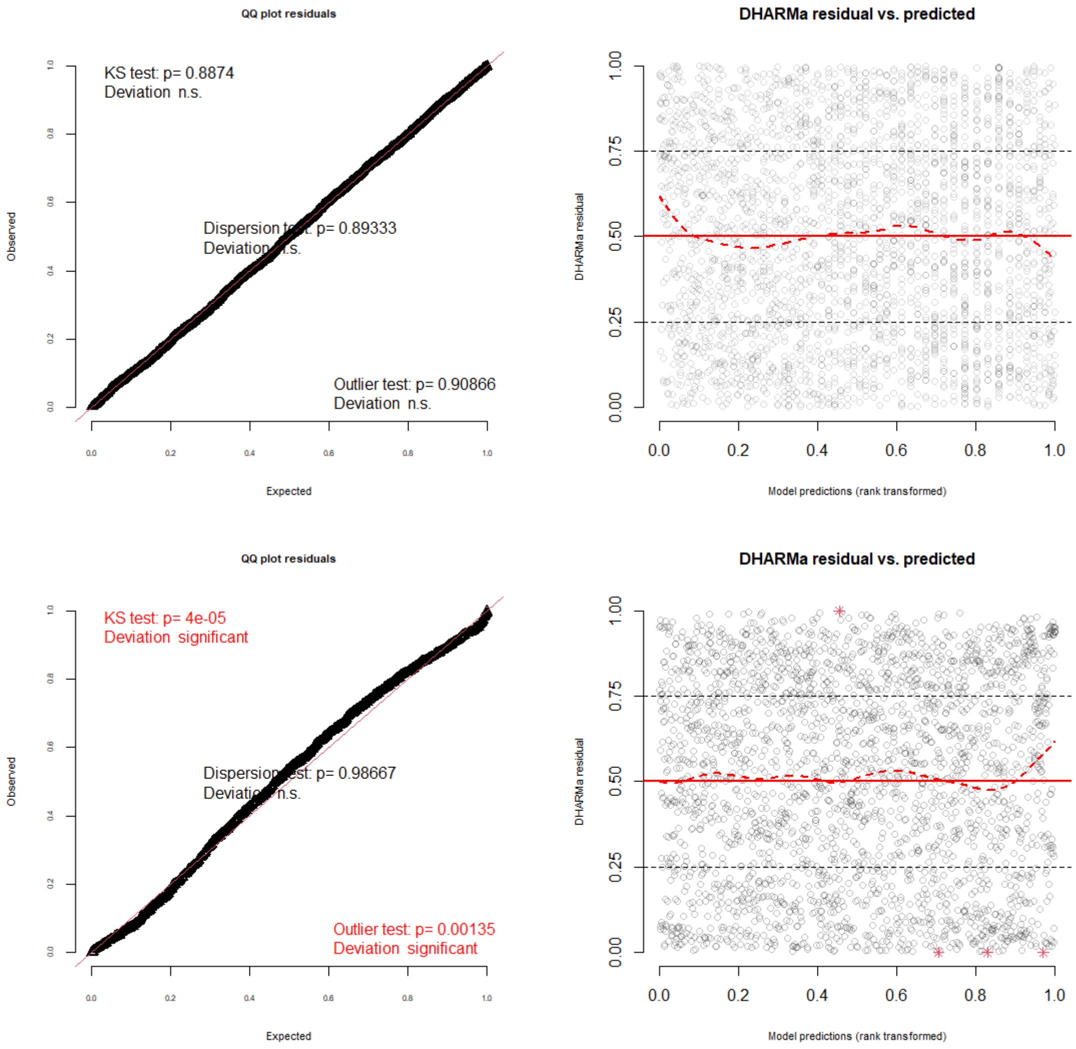

The figure shows the simulated quantile residuals for the model diagnostics. The upper two panels are for the binomial model. The lower two panels are for the normal model. The left-hand panels show the quantile-quantile plot. The right-hand panels show the residuals vs the predictions. Fairly small departures from the straight line are detected for the normal model. A somewhat asymmetric confidence band for the average trend, well within the limits (dashed horizontals) are shown in the right-hand plots.

Our impression is that, while not perfect and not explaining most of the variance, no major departures from the analysis assumptions have been detected, so the final models look reliable.

Sección S7 Fuentes de Alimentos Ultraprocesados

Figura G223 Distribución de Grupos de Alimentos que proporcionan Nova 4 (Ponderada)

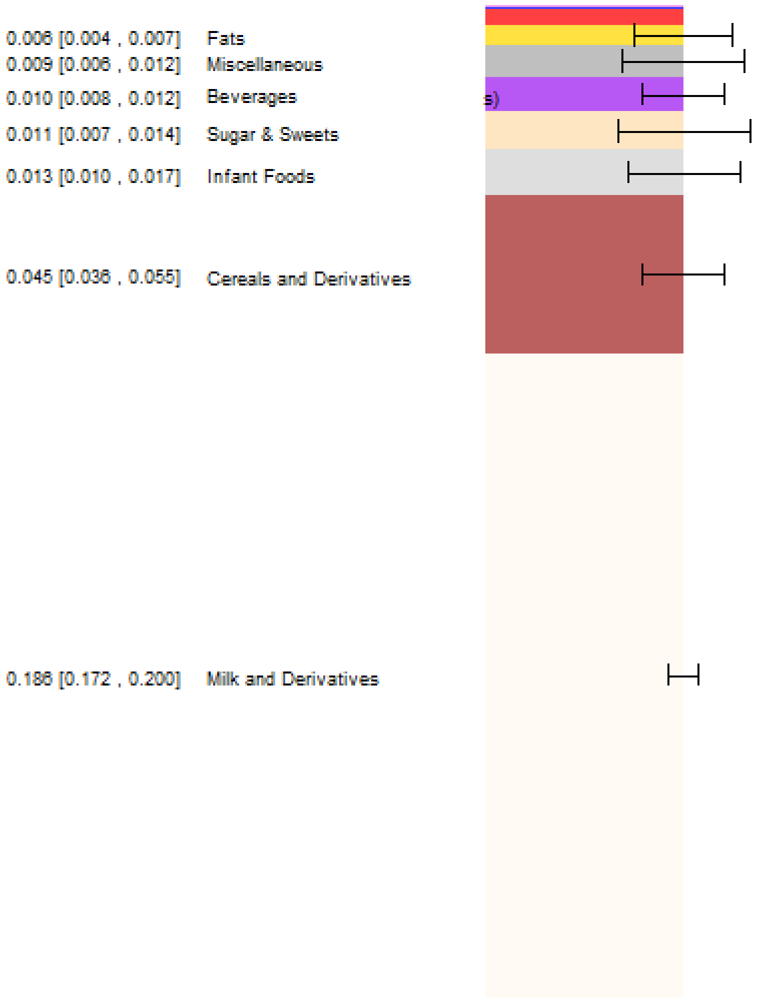

The bars represent the fraction of total energy intake provided by NOVA 4 foods in each food group (each color is a food group, only the top groups are shown). All the bars together add up to the numbers at the bottom of the figure, the fraction of total energy provided by NOVA 4 groups. The numbers on the left represent the weighted fraction estimates, with their 95% confidence intervals within brackets. The error bars on the right side of each bar depict those CI.

Sección S8 Tablas Suplementarias

Tabla S801 Contribución Energética Promedio, Calorías

Contribuciones de energía absoluta expresadas como calorías consumidas diariamente per cápita para cada categoría Nova:

Grupo	N	Calorías Nova 1	Calorías Nova 2	Calorías Nova 3	Calorías Nova 4 AUP
Total	2887	492 [467 a 517]	136 [121 a 151]	63 [55 a 71]	279 [258 a 300]
Sexo Masculino	1457	509 [474 a 543]	138 [120 a 156]	72 [61 a 83]	290 [259 a 321]
Sexo Femenino	1430	474 [444 a 504]	133 [111 a 156]	54 [46 a 61]	267 [244 a 290]
Edad 06-11m	591	218 [187 a 249]	54 [44 a 64]	16 [12 a 20]	168 [138 a 198]
Edad 12-23m	1168	477 [453 a 501]	133 [104 a 162]	64 [53 a 74]	250 [225 a 274]
Edad 24-35m	1128	651 [608 a 694]	182 [160 a 204]	87 [76 a 99]	364 [325 a 403]
Ámbito Lima Metro	749	483 [418 a 548]	109 [85 a 134]	55 [45 a 65]	323 [283 a 363]
Ámbito Urbano	1176	499 [471 a 527]	153 [130 a 176]	72 [60 a 85]	296 [266 a 326]
Ámbito Rural	962	485 [445 a 526]	127 [109 a 146]	48 [37 a 60]	153 [117 a 188]
Periodo 2008	68	460 [354 a 566]	118 [74 a 162]	121 [48 a 195]	246 [210 a 283]
Periodo 2009	483	457 [406 a 507]	144 [110 a 177]	59 [49 a 68]	267 [227 a 306]
Periodo 2010	172	434 [387 a 481]	136 [102 a 170]	54 [41 a 67]	203 [162 a 243]
Periodo 2015	558	538 [488 a 588]	125 [104 a 145]	53 [42 a 63]	335 [269 a 401]
Periodo 2016	643	558 [527 a 589]	153 [133 a 173]	73 [63 a 84]	342 [296 a 388]
Periodo 2019	963	563 [529 a 598]	115 [98 a 131]	54 [47 a 62]	282 [256 a 307]
Pobreza 20%+	1700	464 [437 a 491]	144 [122 a 166]	66 [55 a 78]	234 [208 a 260]
Pobreza <20%	1187	535 [486 a 583]	124 [107 a 140]	58 [50 a 65]	348 [313 a 383]

Calorías/Persona/Día
Promedios aritméticos con sus Intervalos de 95% de confianza entre corchetes.
AUP: Alimentos Ultraprocesados.

Tabla S811 Distribución de la Población según Encuestas y Covariables

Covariable	Categoría	Total	MONIN	VIANEV
Dominio	Lima Metropolitana	29.2 [24.2 a 34.2]	30.9 [22.8 a 38.9]	26.6 [23.8 a 29.5]
Dominio	Urbano	53.1 [48.4 a 57.8]	51.3 [45.1 a 57.4]	55.8 [48.5 a 63.1]
Dominio	Rural	17.8 [15.2 a 20.3]	17.9 [14.1 a 21.6]	17.6 [14.7 a 20.5]
Sexo	femenino	49.2 [44.6 a 53.8]	49.8 [42.9 a 56.6]	48.3 [43.2 a 53.3]
Sexo	masculino	50.8 [46.3 a 55.3]	50.2 [43.5 a 57]	51.7 [46.8 a 56.7]
Edad	6-11 meses	21.5 [18 a 25.1]	22.7 [17 a 28.4]	19.8 [17.2 a 22.3]
Edad	12-17 meses	18.7 [16.1 a 21.2]	18.2 [14.3 a 22.1]	19.4 [17.1 a 21.7]
Edad	18-23 meses	19.3 [16.8 a 21.8]	18.3 [14.5 a 22.1]	20.7 [18.2 a 23.2]
Edad	24-29 meses	19.5 [16.8 a 22.2]	19.5 [15.4 a 23.5]	19.7 [16.7 a 22.6]
Edad	30-35 meses	21 [18.2 a 23.8]	21.4 [17.1 a 25.7]	20.5 [17.4 a 23.5]
Trimestre	Ene-Mar	17.7 [13 a 22.5]	25.3 [18.1 a 32.4]	6.3 [1.1 a 11.5]
Trimestre	Abr-Jun	28.2 [21.6 a 34.9]	28.2 [17.5 a 38.9]	28.2 [24 a 32.5]
Trimestre	Jul-Set	22.8 [19.5 a 26.1]	17.1 [12.2 a 22]	31.4 [27.5 a 35.3]
Trimestre	Oct-Dic	31.3 [25.2 a 37.3]	29.4 [20.8 a 37.9]	34.1 [26.3 a 41.9]
Pobreza	0-14%	24.4 [19.9 a 29]	18.4 [12.2 a 24.5]	33.6 [27.1 a 40.2]
Pobreza	15-19%	14.8 [9.9 a 19.7]	13.3 [5.8 a 20.7]	17.2 [12.2 a 22.2]
Pobreza	20-39%	35.4 [28.6 a 42.1]	38.9 [28.7 a 49.2]	29.9 [23.1 a 36.7]
Pobreza	40-90%	25.4 [20.9 a 29.9]	29.4 [23.3 a 35.6]	19.3 [12.9 a 25.7]

Versión Estratificada y Ponderada de la Tabla 1
Distribución de Covariables, Proporciones Ponderadas, Total y según Encuestas

Tabla S812 Indicadores de Consumo según Encuesta, Procesamiento y Covariables

Encuesta	Grupo	N	Contribución NOVA 1 %	Contribución NOVA 2 %	Contribución NOVA 3 %	Contribución NOVA 4 AUP %	Consumido-res AUP %
MONIN	Total	723	52 [50 a 54]	14 [13 a 16]	7 [6 a 8]	27 [24 a 29]	83 [80 a 87]
	Sexo Masculino	352	52 [49 a 55]	14 [12 a 15]	8 [7 a 9]	27 [23 a 30]	83 [78 a 89]
	Sexo Femenino	371	52 [49 a 55]	15 [13 a 17]	6 [5 a 7]	27 [23 a 30]	84 [79 a 89]
	Edad 06-11 meses	152	50 [43 a 57]	14 [11 a 17]	4 [3 a 6]	31 [23 a 40]	76 [68 a 84]
	Edad 12-23 meses	281	52 [48 a 55]	15 [13 a 17]	8 [7 a 10]	25 [22 a 28]	86 [80 a 92]
	Edad 24-35 meses	290	53 [50 a 56]	14 [12 a 16]	7 [6 a 8]	25 [22 a 28]	86 [80 a 92]
	Ámbito Lima Metro	141	49 [44 a 54]	12 [9 a 14]	6 [5 a 8]	33 [26 a 40]	89 [83 a 96]
	Ámbito Urbano	438	50 [48 a 52]	15 [13 a 17]	8 [6 a 10]	27 [24 a 29]	88 [84 a 92]
	Ámbito Rural	144	63 [57 a 68]	17 [14 a 20]	5 [3 a 7]	15 [10 a 20]	60 [46 a 75]
	Pobreza 20%+	525	53 [50 a 56]	16 [14 a 18]	7 [6 a 9]	24 [20 a 28]	80 [75 a 86]
	Pobreza <20%	198	51 [48 a 54]	12 [10 a 13]	6 [5 a 7]	31 [28 a 35]	90 [86 a 95]
	Periodo 2008	68	49 [45 a 54]	13 [10 a 16]	11 [6 a 16]	27 [22 a 32]	87 [80 a 95]
	Periodo 2009	483	51 [48 a 55]	14 [12 a 16]	7 [6 a 8]	28 [24 a 32]	83 [78 a 88]
	Periodo 2010	172	55 [51 a 59]	16 [13 a 18]	6 [5 a 8]	23 [19 a 27]	83 [74 a 91]
VIANEV	Total	2164	55 [54 a 56]	12 [11 a 12]	6 [5 a 6]	27 [26 a 29]	91 [89 a 93]
	Sexo Masculino	1105	55 [53 a 57]	12 [11 a 13]	6 [5 a 6]	27 [25 a 29]	90 [88 a 93]
	Sexo Femenino	1059	55 [53 a 57]	12 [11 a 13]	6 [5 a 7]	28 [25 a 30]	91 [88 a 94]
	Edad 06-11 meses	439	58 [54 a 62]	11 [9 a 13]	4 [2 a 5]	28 [24 a 32]	79 [74 a 84]
	Edad 12-23 meses	887	57 [55 a 59]	12 [11 a 13]	6 [5 a 6]	26 [24 a 28]	93 [90 a 95]
	Edad 24-35 meses	838	52 [50 a 54]	12 [11 a 13]	7 [6 a 7]	29 [27 a 31]	95 [93 a 97]
	Ámbito Lima Metro	608	53 [51 a 55]	11 [9 a 12]	5 [4 a 6]	31 [28 a 33]	94 [92 a 96]
	Ámbito Urbano	738	53 [52 a 55]	12 [11 a 13]	6 [5 a 7]	29 [26 a 32]	93 [90 a 95]
	Ámbito Rural	818	63 [60 a 66]	13 [12 a 15]	6 [5 a 7]	18 [16 a 21]	79 [75 a 84]
	Pobreza 20%+	1175	57 [55 a 59]	13 [12 a 14]	6 [5 a 7]	24 [22 a 26]	87 [84 a 90]
	Pobreza <20%	989	53 [51 a 55]	11 [10 a 12]	5 [4 a 6]	31 [28 a 33]	94 [92 a 96]
	Periodo 2015	558	53 [51 a 56]	12 [11 a 13]	6 [4 a 7]	29 [26 a 33]	92 [89 a 95]
	Periodo 2016	643	53 [51 a 56]	13 [12 a 14]	6 [5 a 7]	28 [25 a 30]	91 [88 a 94]
	Periodo 2019	963	59 [56 a 61]	11 [10 a 12]	5 [5 a 6]	25 [23 a 28]	88 [86 a 91]

Versión Estratificada y Ponderada de la Tabla 2

Distribución de Indicadores de Consumo, según Encuestas

Tabla S813 Contribución Energética Promedio según Encuesta, Procesamiento y Grupo

Encuesta	Nova	Grupo de Alimentos	Estimado	Límite Inferior	Límite Superior
MONIN	No|Mín Procesados	Cereales y derivados	20,4	18,5	22,2
		Verduras, Hortalizas y derivados	0,9	0,7	1
		Frutas y derivados	8,2	7,2	9,2
		Pescados y Mariscos	0,6	0,4	0,9
		Carnes y derivados	4,5	3,8	5,2
		Leches y derivados	2,6	1,8	3,5
		Huevos y derivados	2,8	2,4	3,2
		Leguminosas y derivados	1,9	1,3	2,5
		Tubérculos, Raíces y derivados	4,7	4	5,5
		Otros	3,2	2,3	4,2
	Ingredientes	Grasas, Aceites y Oleaginosas	8	5,8	10,1
		Productos Azucarados	7,1	6	8,3
		Otros	0,2	0,1	0,4
	Procesados	Cereales y derivados	5,1	4,5	5,7
		Leches y derivados	0,5	0,3	0,7
		No es un Alimento Simple	1,4	0,6	2,3
		Otros	0,2	0,1	0,2
	Ultraprocesados	Cereales y derivados	4,7	3	6,3
		Leches y derivados	17,7	15,4	20
		Productos Azucarados	1,2	0,6	1,8
		Otros	4,1	3,1	5
VIANEV	No|Mín Procesados	Cereales y derivados	21,1	20,2	22
		Verduras, Hortalizas y derivados	0,9	0,8	1
		Frutas y derivados	9,1	8,4	9,8
		Pescados y Mariscos	1,2	0,9	1,6
		Carnes y derivados	5,3	4,9	5,7
		Leches y derivados	1	0,6	1,3
		Huevos y derivados	3,7	3,4	3,9
		Leguminosas y derivados	1,8	1,5	2,1
		Tubérculos, Raíces y derivados	6,7	6	7,3
		Otros	1,1	0,7	1,4
	Ingredientes	Grasas, Aceites y Oleaginosas	3,9	3,4	4,4
		Productos Azucarados	8	7,3	8,7
		Otros	0,4	0,2	0,7
	Procesados	Cereales y derivados	4,6	4,1	5,1
		Leches y derivados	0,7	0,6	0,9
		No es un Alimento Simple	0	0	0
		Otros	0,3	0,1	0,4
	Ultraprocesados	Cereales y derivados	4,3	3,9	4,8
		Leches y derivados	19,8	18,3	21,2
		Productos Azucarados	0,9	0,7	1,2
		Otros	5,2	4,6	5,8

Versión Estratificada y Ponderada de la Tabla 3

Tabla S814 Modelamiento de Indicadores según Encuesta y Covariables

Encuesta	Modelo	Término	Pendiente	Error Estándar	Valor_p
MONIN	binomial	Intercepto	0,680	0,876	0,438
		Edad, logaritmo	0,800	0,276	0,004
		Pobreza distrital, %	-0,037	0,007	0,000
	normal	Intercepto	11,248	2,527	0,000
		Edad, m	0,133	0,064	0,039
		Edad, logaritmo	-3,124	1,282	0,016
		Abril-Junio	0,878	0,322	0,007
		Julio-Setiembre	0,452	0,284	0,113
		Octubre-Diciembre	0,218	0,250	0,384
VIANEV	binomial	Intercepto	-0,615	0,561	0,273
		Edad, logaritmo	1,316	0,195	0,000
		Pobreza distrital, %	-0,029	0,006	0,000
	normal	Intercepto	9,655	1,578	0,000
		Edad, m	0,142	0,040	0,001
		Edad, logaritmo	-2,612	0,806	0,001
		Abril-Junio	0,154	0,434	0,723
		Julio-Setiembre	0,113	0,431	0,794
		Octubre-Diciembre	0,289	0,483	0,551

Versión Estratificada y Ponderada de la Tabla 4
Modelos finales, según Encuestas

Sección S9 Fragmentos Actualizados del Programa

La función FMO0 del programa original usado en la prepublicación fue modificada para el artículo en la RPMESP, retirando los términos de antropometría, quedando como sigue:

```
FMO0=function(k){
# modelaje: barrido bootstrap de predictores, sin antropometria
	require(survey)
	# generación de muestra
	ZTOT=
			FRS1(LRS0)%>%
			left_join(y=LDAT$UTOT,by=c("IDESP","IDCLU","IDNIP"))%>%
			mutate(IDCLU=IDCLX,IDNIP=IDNIX)%>%
			select(
				E4RB,E4RS,
				EDM,BSEX,IDDOM,PPOB,DAEN,AAEN,
				FTEN,TREN,DSEN,DMEN,SDEN,EDML,
				IDCLU,IDNIP,IDESP,WPOND
			)%>%
			drop_na()
			# HFAZ,WFHZ,HFA2,WFH2,
	x=LDAT$STOT
	#s=update(object=x,data=ZTOT)
	S=
		survey::svydesign(
			ids=~IDCLU+IDNIP,strata=~IDESP,
			weights=~WPOND,nest=TRUE,data=ZTOT
		)

	# componente binomial
	# . barrido con transformaciones sin interacciones
	m=
		survey::svyglm(
			formula=
				E4RB~
				EDM+BSEX+IDDOM+PPOB+DAEN+AAEN+
				FTEN+DSEN+DMEN+SDEN+EDML,
			design=S,family=quasibinomial
		)
		# +HFAZ+WFHZ+HFA2+WFH2
	m=MASS::stepAIC(object=m,trace=0,direction="backward")
	# require(survey)
	# . barrido en remanente, con interacciones
	m=
		update(
			object=m,
			formula=E4RB~.+EDM:BSEX+IDDOM:PPOB+DAEN:AAEN
		)
	# +HFAZ:WFHZ
	m=MASS::stepAIC(object=m,trace=0,direction="backward")
	# require(survey)
	# . acumulación de selecciones
	s=as.data.frame(summary(m)$coefficients[,c(1,4)])
	names(s)=c("best","pval")
	s$term=rownames(s);rownames(s)=NULL;s$krep=k
	ob=s	#OB[[k]]=s	# as.character(m$call)[2]
```

```
	# componente normal
	# . barrido con transformaciones sin interacciones
	m=
		survey::svyglm(
			formula=
				E4RS~
				EDM+BSEX+IDDOM+PPOB+DAEN+AAEN+
				FTEN+DSEN+DMEN+SDEN+EDML,
			design=subset(S,E4RB==1),family=gaussian
		)
	# +HFAZ+WFHZ+HFA2+WFH2
	m=MASS::stepAIC(object=m,trace=0,direction="backward")
	# require(survey)
	# . barrido en remanente, con interacciones
	m=
		update(
			object=m,
			formula=E4RS~.+EDM:BSEX+IDDOM:PPOB+DAEN:AAEN
		)
	# +HFAZ:WFHZ
	m=MASS::stepAIC(object=m,trace=0,direction="backward")
	# require(survey)

	# . acumulación de selecciones
	s=as.data.frame(summary(m)$coefficients[,c(1,4)])
	names(s)=c("best","pval")
	s$term=rownames(s);rownames(s)=NULL;s$krep=k
	og=s	#OG[[k]]=s	# as.character(m$call)[2]
	return(list(ob,og))
}
```

Sección SA Referencias Adicionales

1. Asociación Benéfica Prisma. Análisis Nutricional de la Dieta según Requerimientos y Adecuación (ANDREA®). Lima, PE: Prisma; 2003.

2. Austin PC, Tu JV. Automated variable selection methods for logistic regression produced unstable models for predicting acute myocardial infarction mortality. J Clin Epidemiol. 2004 Nov;57(11):1138–46.

3. Box GEP, Cox DR. An Analysis of Transformations. Journal of the Royal Statistical Society Series B (Methodological) [Internet]. 1964 [cited 2018 Jul 29];26(2):211–52. Available from: http://www.jstor.org/stable/2984418

4. Campos M. btools: Basic collection of tools for analytical work [Internet]. Lima, PE: UPCH; 2021 [cited 2021 Jul 25]. Available from: https://github.com/vipermcs/btools

5. Campos-Sánchez M, Ricaldi-Sueldo R, Miranda-Cuadros M, Equipo MONIN. Diseño del Monitoreo Nacional de Indicadores Nutricionales (MONIN), Peru 2007-2010]. Rev Peru Med Exp Salud Publica [Internet]. 2011 Jun;28(2):210–21. Available from: https://scielosp.org/scielo.php?script=sci_arttext&pid=S1726-46342011000200007&lng=en&nrm=iso&tlng=en

6. Dunn PK, Smyth GK. Randomized Quantile Residuals. Journal of Computational and Graphical Statistics [Internet]. 1996 [cited 2021 May 17];5(3):236–44. Available from: https://www.jstor.org/stable/1390802

7. Food and Agriculture Organization (FAO). Human energy requirements - Report of a Joint FAO/WHO/UNU Expert Consultation Rome, 17–24 October 2001 [Internet]. Rome, IT: FAO; 2004. Report No.: 1. Available from: http://www.fao.org/publications/card/es/c/e1faed04-3a4c-558d-8ec4-76a1a7323dcc/

8. Food and Agriculture Organization (FAO). Codex Alimentarius / Codex General Standard for Food Additives (GSFA) Online Database [Internet]. Rome, IT: FAO; 2021 [cited 2023 Dec 16]. Available from: https://www.fao.org/fao-who-codexalimentarius/codex-texts/dbs/gsfa/en/

9. Fox J, Weisberg S. An R Companion to Applied Regression. Los Angeles, CA, US: SAGE Publications; 2018. 608 p.

10. Harrell FE. Regression modeling strategies: with applications to linear models, logistic and ordinal regression, and survival analysis. Second edition. New York, NY, USA: Springer; 2015. 582 p. (statistics).

11. Hartig F. DHARMa | Diagnostics for HierArchical Regression Models [Internet]. 2022 [cited 2021 Nov 28]. Available from: http://florianhartig.github.io/DHARMa/

12. Hawkins DM, Weisberg S. Combining the Box-Cox power and generalised log transformations to accommodate nonpositive responses in linear and mixed-effects linear models. South African Statistical Journal [Internet]. 2017 Sep 30 [cited 2023 Dec 17];51(2):317–28. Available from: https://www.journals.ac.za/sasj/article/view/5215

13. Institute of Medicine (U.S.), Institute of Medicine (U.S.), editors. Dietary reference intakes for energy, carbohydrate, fiber, fat, fatty acids, cholesterol, protein, and amino acids [Internet].

Washington, D.C: National Academies Press; 2005. 1331 p. Available from: https://www.nap.edu/catalog/10490/dietary-reference-intakes-for-energy-carbohydrate-fiber-fat-fatty-acids-cholesterol-protein-and-amino-acids

14. Li J, Valliant R. Linear Regression Influence Diagnostics for Unclustered Survey Data. Journal of Official Statistics. 2011;27(1):99–119.

15. Li J, Valliant R. Linear Regression Diagnostics in Cluster Samples. Journal of Official Statistics [Internet]. 2015 Feb 28 [cited 2023 Nov 29];31(1):61–75. Available from: https://sciendo.com/article/10.1515/jos-2015-0003

16. Liao D. Collinearity Diagnostics for Complex Survey Data [Internet] [Doctor of Phylosophy]. [College Park, MD]: University of Maryland; 2010 [cited 2023 Nov 29]. Available from: http://hdl.handle.net/1903/10881

17. Liao D, Valliant R. Variance inflation factors in the analysis of complex survey data. Survey Methodology [Internet]. 2012 Jun;38(1):53–62. Available from: https://www150.statcan.gc.ca/n1/pub/12-001-x/2012001/article/11685-eng.pdf

18. Lohr SL. Sampling: design and analysis. 2nd ed. Boston, Mass: Brooks/Cole; 2010. 596 p.

19. Lumley T. Complex surveys: a guide to analysis using R. Hoboken, N.J: John Wiley; 2010. 276 p. (Wiley series in survey methodology).

20. McCullagh P, Nelder JA. Generalized Linear Models. 2nd ed. Boca Raton, FL: Chapman and Hall/CRC; 1989. 532 p.

21. Miranda Cuadros MY. Criterios de Focalización de Zonas de Intervención en Vitamina A, basado en las Características del Patrón Alimentario de Niños de 6 a 35 meses de Edad [Maestro en Salud Pública con mención en Epidemiología]. [Lima, PE]: Universidad Nacional Federico Villareal; 2014.

22. Miranda M, Lescano G, Montes C, Segura L, Marín C. Tabla de medidas caseras para la programación y evaluación de regimenes alimenticios (con Laminario y Diskette). Lima, PE: Prisma; 1996 Dic.

23. Miranda-Cuadros M, Campos-Sánchez M. Informe de Resultados de la Ingesta de Energía y otros nutrientes en niños de 6 a 35 meses de edad según MONIN 2008-2010 [Internet]. Lima, PE: INS/CENAN; 2012. Available from: https://web.ins.gob.pe/sites/default/files/Archivos/cenan/van/vigilacia_poblacion/Informe%20Ingesta%20de%20nutrientes%20en%20NI%C3%B1os%202008-2010.pdf

24. Monteiro CA, Cannon G, Levy RB, Moubarac JC, Louzada ML, Rauber F, et al. Ultra-processed foods: what they are and how to identify them. Public Health Nutr. 2019 Feb 12;1–6.

25. Monteiro CA, Cannon G, Lawrence M, Louzada ML da C, Machado PP. Ultra-processed foods, diet quality, and health using the NOVA classification system [Internet]. Rome, IT: FAO; 2019 [cited 2023 Dec 16]. Available from: https://www.fao.org/documents/card/en?details=CA5644EN/

26. Pan American Health Organization (PAHO), World Health Organization (WHO). Ultra-processed food and drink products in Latin America: Sales, sources, nutrient profiles, and policy implications [Internet]. Washington, DC, USA: PAHO/WHO; 2019 [cited 2023 Mar 19]. Available from: https://iris.paho.org/handle/10665.2/51094

27. Perú, Instituto Nacional de Estadística e Informática (INEI). Perú: Estimaciones y Proyecciones de Población, 1950-2050 [Internet]. Lima, PE: INEI; 2009 Mar. (Boletín de Análisis Demográfico). Report No.: 36. Available from: http://proyectos.inei.gob.pe/web/biblioineipub/bancopub/Est/Lib0845/index.htm

28. Perú, Instituto Nacional de Estadística e Informática (INEI). PERÚ: Estimaciones y Proyecciones de Población por Departamento, Sexo y Grupos Quinquenales de Edad 1995-2025 [Internet]. Lima, PE: INEI; 2009 Oct. (Boletín de Análisis Demográfico). Report No.: 37. Available from: http://proyectos.inei.gob.pe/web/biblioineipub/bancopub/Est/Lib0846/index.htm

29. Perú, Instituto Nacional de Estadística e Informática (INEI). Perú: Mapa de Pobreza Provincial y Distrital 2009 - El enfoque de la pobreza monetaria [Internet]. Lima, PE: INEI; 2010 Oct. Available from: https://www.inei.gob.pe/media/MenuRecursivo/publicaciones_digitales/Est/Lib1261/

30. Perú, Instituto Nacional de Estadística e Informática (INEI). Mapa de Pobreza Provincial y Distrital 2013 [Internet]. Lima, PE: INEI; 2015 Sep. Available from: https://www.inei.gob.pe/media/MenuRecursivo/publicaciones_digitales/Est/Lib1261/

31. Perú, Instituto Nacional de Estadística e Informática (INEI). Mapa de pobreza monetaria provincial y distrital 2018 [Internet]. Lima, PE: INEI; 2020 Feb. Available from: https://www.inei.gob.pe/media/MenuRecursivo/publicaciones_digitales/Est/Lib1718/Libro.pdf

32. Perú, Instituto Nacional de Salud (INS). Informe Final - Encuesta: Vigilancia Alimentaria y Nutricional por etapas de vida – VIANEV - niños menores de 36 meses - 2015 [Internet]. Lima, PE: INS/CENAN; 2018. Available from: https://web.ins.gob.pe/sites/default/files/Archivos/cenan/van/vigilacia_poblacion/Informe_VIANEV_Ninos_2015.pdf

33. Perú, Instituto Nacional de Salud (INS). Informe Técnico: Estado nutricional, consumo aparente de lactancia materna y consumo de alimentos en niños menores de 3 años de la encuesta Vigilancia Alimentaria y Nutricional por etapas de vida - VIANEV 2016. [Internet]. Lima, PE: INS/CENAN; 2021. Available from: https://web.ins.gob.pe/sites/default/files/Archivos/cenan/van/vigilacia_poblacion/Informe_VIANEV_Ninos_2015.pdf

34. Perú, Instituto Nacional de Salud (INS). Informe Técnico: Estado nutricional y consumo de alimentos del niño menor de 3 años de la Encuesta Vigilancia Alimentaria y Nutricional por Etapas de Vida - VIANEV 2019 [Internet]. 2023 Sep [cited 2023 Sep 19]. Available from: https://www.gob.pe/institucion/ins/informes-publicaciones/4202369-informe-tecnico-estado-nutricional-y-consumo-de-alimentos-del-nino-menor-de-3-anos-de-la-encuesta-vigilancia-alimentaria-y-nutricional-por-etapas-de-vida-vianev-2019

35. Perú, Instituto Nacional de Salud (INS), Centro Nacional de Alimentación y Nutrición (CENAN). Monitoreo Nacional de Indicadores Nutricionales (MONIN), CENAN 2007-2010 [Internet]. Plataforma Nacional de Datos Abiertos; 2023 [cited 2023 Nov 24]. Available from: https://www.datosabiertos.gob.pe/dataset/monitoreo-nacional-de-indicadores-nutricionales-monin-cenan-2007-2010

36. Perú, Instituto Nacional de Salud (INS), Centro Nacional de Alimentación y Nutrición (CENAN). Encuesta VIANEV 2017-2018. Estado nutricional en niños menores de 3 años [Internet]. Plataforma Nacional de Datos Abiertos; 2023 [cited 2023 Nov 24]. Available from:

https://www.datosabiertos.gob.pe/dataset/encuesta-vianev-2017-2018-estado-nutricional-en-ni%C3%B1os-menores-de-3-a%C3%B1os-ins-cenan

37. Perú, Instituto Nacional de Salud (INS), Centro Nacional de Alimentación y Nutrición (CENAN). Encuesta VIANEV niños 2016. Estado nutricional, consumo de LM y consumo de alimentos. [Internet]. Plataforma Nacional de Datos Abiertos; 2023 [cited 2023 Nov 24]. Available from: https://www.datosabiertos.gob.pe/dataset/encuesta-vianev-ni%C3%B1os-2016-estado-nutricional-consumo-de-lm-y-consumo-de-alimentos-ins-cenan

38. Perú, Instituto Nacional de Salud (INS), Centro Nacional de Alimentación y Nutrición (CENAN). Monitoreo Nacional de Indicadores Nutricionales (MONIN), CENAN 2007 y 2008 [Internet]. Plataforma Nacional de Datos Abiertos; 2023 [cited 2023 Nov 24]. Available from: https://www.datosabiertos.gob.pe/dataset/monitoreo-nacional-de-indicadores-nutricionales-monin-cenan-2007-y-2008

39. Perú, Instituto Nacional de Salud (INS), Centro Nacional de Alimentación y Nutrición (CENAN). Vigilancia Alimentaria y Nutricional - VIANEV - niños menores de 36 meses - 2015 [Internet]. Plataforma Nacional de Datos Abiertos; 2023 [cited 2023 Nov 24]. Available from: https://www.datosabiertos.gob.pe/dataset/vigilancia-alimentaria-y-nutricional-vianev-ni%C3%B1os-menores-de-36-meses-2015

40. Perú, Instituto Nacional de Salud (INS), Centro Nacional de Alimentación y Nutrición (CENAN). Encuesta VIANEV 2019. Estado nutricional y consumo de alimentos del niño menor de 5 años de la Encuesta Vigilancia Alimentaria y Nutricional por Etapas de Vida [Internet]. Plataforma Nacional de Datos Abiertos; 2023 [cited 2023 Nov 24]. Available from: https://www.datosabiertos.gob.pe/dataset/encuesta-vianev-2019-estado-nutricional-y-consumo-de-alimentos-del-ni%C3%B1o-menor-de-5-a%C3%B1os-de

41. Peru, Instituto Nacional de Salud, Centro Nacional de Alimentación y Nutrición (CENAN), editor. Tablas Peruanas de Composición de Alimentos. Lima, PE: INS/CENAN; 2017.

42. R Core Team. R: A language and environment for statistical computing [Internet]. Vienna, Austria: R Foundation for Statistical Computing; 2023 [cited 2013 Feb 8]. Available from: http://www.R-project.org

43. Rizopoulos D. bootStepAIC: Bootstrap stepAIC [Internet]. 2022 [cited 2023 Jan 14]. Available from: https://CRAN.R-project.org/package=bootStepAIC

44. Tukey J. Exploratory Data Analysis. Reading, MA: Addison-Wesley; 1977.

45. World Health Organization (WHO). WHO child growth standards: length/height-for-age, weight-for-age, weight-for-length, weight-for-height and body mass index-for-age ; methods and development [Internet]. Geneva: WHO Press; 2006. 312 p. Available from: https://www.who.int/publications-detail-redirect/924154693X
