# Supplementary material for: Energy contribution from ultra processed foods in peruvian children
Source: Rev Peru Med Exp Salud Publica. 2025 Sep 29;42(3):240–51. doi: 10.17843/rpmesp.2025.423.14339 (PMC12679980; doi:10.17843/rpmesp.2025.423.14339)
Supplement: Translation

# Translation: Contribución energética de los alimentos ultraprocesados en niños peruanos

**Authors:** Marianella Miranda-Cuadros, Miguel Campos-Sánchez, Gustavo Cediel Giraldo, María Laura da Costa Louzada, Joaquín Alejandro Marrón-Ponce

**Affiliations:** 1 Instituto Nacional de Salud, Lima, Perú.; 2 Universidad Peruana Cayetano Heredia, Lima, Perú.; 3 Universidad de Antioquía, Medellín, Colombia.; 4 Universidad de Sao Paulo, Sao Paulo, Brasil.; 5 Instituto Nacional de Salud Pública, Cuernavaca, México.

**Keywords:** Perú, Niños, Consumo de Alimentos, Energía, Alimentos Ultraprocesados

## Abstract

**Objetivo.:**

Evaluar la contribución energética de alimentos ultraprocesados (AUP) y su asociación con covariables sociales y demográficas en niños entre 6 y 35 meses de edad, a partir de encuestas nacionales realizadas en 2008‒2010, 2015‒2016 y 2019.

**Materiales y métodos.:**

Las encuestas utilizaron muestras aleatorias estratificadas multietápicas. Se aplicaron recordatorios de 24 horas en días aleatorios por participante, mediante el método de pasos múltiples modificado, con apoyo de ayudas visuales y balanzas. Los AUP pertenecieron al grupo Nova 4. Dada la distribución bimodal, las covariables se analizaron mediante dos modelos: binomial para el porcentaje de consumidores de AUP y normal para la contribución energética promedio, solo entre consumidores de AUP. Las estimaciones y los modelos fueron ajustados según el diseño muestral.

**Resultados.:**

Se incluyó 2887 niños. Los AUP contribuyeron con el 27% (IC 95%: 25 a 29) de la ingesta total de energía y fueron consumidos por 86% (84 a 89) de niños. La principal contribución energética de los AUP provino del grupo leche y derivados (19% [17 a 20]) y cereales (5% [4 a 6]). Las covariables asociadas al consumo fueron la edad, el trimestre calendario y la pobreza. No se encontraron asociaciones con el sexo ni con el año de la encuesta.

**Conclusiones:**

. En niños de 6 a 35 meses en el Perú, los AUP aportaron en promedio el 27% de la energía total, y fueron consumidos por el 86%. Las principales fuentes fueron los grupos de leche y derivados, y cereales. El consumo de AUP se asoció con la edad, la pobreza y el trimestre calendario.

## INTRODUCCIÓN

El sobrepeso y la obesidad representan retos actuales a la salud mundial ^1^. Son determinantes claros de enfermedades crónicas graves ^2^^,^^3^. Su prevalencia global está en aumento tanto en jóvenes como en adultos ^4^^,^^5^, y se encuentra vinculada con la múltiple carga de la malnutrición a lo largo del ciclo de vida ^6^. Aunque en menor proporción, también están presentes en lactantes y niños ^7^^,^^8^. En el Perú, se ha reportado una prevalencia del 9% para ambas condiciones en niños menores de cinco años ^9^^,^^10^.

Uno de los principales determinantes del sobrepeso y la obesidad es el consumo de alimentos ultraprocesados (AUP) ^11^^-^^13^. Según la Organización Mundial de la Salud (OMS), los AUP son «formulaciones industriales elaboradas principal o totalmente a partir de sustancias derivadas de componentes de los alimentos, además de los aditivos usados para imitar e intensificar las cualidades sensoriales de los productos sin procesar o mínimamente procesados y los platos, y comidas preparados con esos productos y con ingredientes culinarios procesados» ^14^. Los AUP incluyen alimentos que, en diverso grado, aportan en exceso energía, azúcares, sodio, grasas trans y saturadas a la dieta ^15^, además de aditivos no calóricos y edulcorantes, los cuales carecen de valor nutricional ^16^. El consumo de AUP se ha asociado con adiposidad en niños mayores ^11^^,^^12^ y con enfermedades cardiovasculares y metabólicas en adultos ^(^^17^^-^^20^.

Cada vez existen más estudios sobre el consumo de AUP en niños pequeños. Una revisión multinacional ^21^ reportó que la ingesta de AUP en niños con edad entre 2 y 5 años representó el 44% de la ingesta energética total en Chile, el 38% en México, el 27% en Argentina, y el 18% en Colombia ^22^^-^^25^. En el Perú, no se han publicado estimaciones previas sobre el consumo de AUP en niños pequeños.

El presente estudio tiene como objetivo estimar la contribución energética de los AUP y su asociación con algunas covariables sociales y demográficas en niños de 6 a 35 meses de edad en el Perú, utilizando dos encuestas nacionales: Monitoreo Nacional de Indicadores Nutricionales (MONIN) ^26^ y Vigilancia Alimentaria y Nutricional por Etapas de Vida (VIANEV) ^27^^-^^29^. Estos resultados serán relevantes para sustentar estrategias y políticas orientadas a promover una alimentación saludable y controlar el sobrepeso y la obesidad.

**MENSAJE CLAVE**

Motivación para realizar el estudio. La obesidad es problema global creciente en niños. Los alimentos ultraprocesados (AUP) son un determinante importante, cuyo nivel de consumo en niños del Perú aún no ha sido reportado.

Principales hallazgos. Los resultados indican que, en niños 6 a 35 meses, los AUP contribuyen con el 27% de la energía consumida, son ingeridos por el 86% de los niños, provienen mayormente de productos lácteos y cereales, y su consumo varía según la edad, la pobreza y la estacionalidad.

Implicancias para la salud pública. Proponemos indicadores para vigilar el consumo de AUP, evaluar si las intervenciones lo reducen e identificar sus determinantes. Sugerimos estudiar los beneficios y riesgos del consumo de leche evaporada en niños.

## MATERIALES Y MÉTODOS

Este artículo presenta un análisis secundario de dos encuestas nacionales transversales y repetidas, con muestreo aleatorio estratificado multietápico, que evaluaron la dieta infantil mediante recordatorio de 24 horas (R24h): MONIN (2008-2010) y VIANEV (2015-2016 y 2019).

### Población y muestra

MONIN incluyó a niños menores de cinco años, dividiendo el país en cinco ámbitos geográficos: Lima Metropolitana (provincias de Lima y Callao), resto de costa (Andes occidentales por debajo de 2000 m s. n. m.), sierra urbana (centros poblados a 2000 m o más s. n. m. con 2000 o más habitantes), sierra rural (centros poblados a 2000 m o más s. n. m. con menos de 2000 habitantes) y selva (Andes orientales debajo de 2000 m s. n. m.); entre noviembre del 2007 y abril del 2010.

VIANEV incluyó menores de tres años, dividiendo el país en tres ámbitos geográficos: Lima Metropolitana, urbano y rural, en tres periodos de recolección: octubre-diciembre de 2015, abril-julio de 2016 y agosto-octubre de 2019.

El universo incluyo a todos los residentes del Perú en las edades y periodos indicados. El diseño muestral fue estratificado y de dos etapas. Los estratos correspondieron a los dominios geográficos, subdivididos según el nivel económico censal. En la primera etapa, el marco muestral fue la lista de conglomerados censales definidos por el Instituto Nacional de Estadística e Informática (INEI). En cada estrato, se seleccionaron conglomerados mediante muestreo aleatorio sin reemplazo, con probabilidad proporcional al tamaño (aproximadamente 100 viviendas por conglomerado) y se asignaron aleatoriamente a las semanas programadas. En la segunda etapa, los equipos de campo enumeraron los hogares elegibles en los conglomerados seleccionados y tomaron una muestra aleatoria simple de hogares sin reemplazo (10 en MONIN y 5 en VIANEV). Los pesos muestrales originales se calcularon como la inversa del producto de las probabilidades de selección en ambas etapas. Estas probabilidades fueron reescaladas a la población proyectada para cada año (material suplementario sección S3).

Para el presente artículo los criterios de inclusión fueron: niños entre 6 y 35 meses de edad, al menos un R24h con energía registrada, puntaje z de talla para la edad e identificación de distrito de residencia. De un total de 3022 niños en ese rango de edad con R24h en los periodos estudiados, se incluyó a 2887.

### Medición de consumo de alimentos

Para evaluar la dieta, se registró la lista completa de alimentos y bebidas consumidos durante las 24 horas previas a la entrevista, aplicando la metodología de pasos múltiples ^30^^)^ modificada mediante la exclusión de alimentos olvidados. El informante fue la persona encargada de la preparación y el servido de los alimentos, siendo generalmente la madre del niño. En los casos en que el niño era beneficiario de Cuna Más, un programa social de guarderías para cuidado diurno ^31^, los responsables del programa fueron entrevistados.

El R24h se aplicó en un día aleatorio. Tras la selección aleatoria de los conglomerados en cada periodo, estos se reordenaron mediante una permutación aleatoria preestablecida en forma de secuencia semanal. Posteriormente, los 10 hogares planificados por semana se reordenaron también mediante una permutación aleatoria de siete días (con tres días adicionales seleccionados mediante muestreo aleatorio simple). La entrevista del R24h se concertó con el hogar dentro de los tres días cercanos al día aleatorio asignado.

La recolección del R24h se apoyó con ayudas visuales (maquetas de alimentos y laminario), balanzas de precisión y tablas de composición de alimentos y equivalencias de medidas caseras para alimentos y preparaciones ^32^^,^^33^. No se registró información sobre lactancia materna ni sobre suplementos alimentarios. La capacitación de los entrevistadores consistió en un taller de 12 días que incluyó dos sesiones piloto en campo, en ambas encuestas.

### Clasificación de alimentos según el procesamiento industrial

Los criterios para la categorización de los alimentos siguieron la clasificación Nova (material suplementario sección S1). Los AUP corresponden a la categoría Nova 4.

Cada alimento o bebida fue clasificado mediante una secuencia computarizada de reglas que asignaron el grupo Nova correspondiente, según datos contenidos en la tabla compilada de composición de alimentos (grupo CENAN/ANDREA: cereales, frutas, etc.), los códigos de alimento y términos clave (requeridos como presentes o ausentes en la descripción). Las reglas fueron definidas por una nutricionista experta en un formato de hoja de cálculo, que fue revisado por un programador para verificar la adecuación del formato (espaciado, uso de mayúsculas, caracteres especiales y expresiones lógicas).

En el material suplementario (tabla S102) se describe las reglas utilizadas por cada grupo de alimentos. Por ejemplo, en el grupo de cereales, se clasificaron como Nova 1 los granos enteros o semillas, harinas, hojuelas y fideos; como Nova 2, la harina de maíz (maicena); como Nova 3, el pan recién horneado y las hojuelas azucaradas precocidas; y como Nova 4, el pan etiquetado y los productos de cereales de diversas marcas comerciales.

Para los productos etiquetados no incluidos en las tablas de composición de alimentos se consultaron sitios web de fabricantes, así como la base de datos Open Food Facts ^34^.

### Medición de covariables

Se incluyeron las siguientes covariables (material suplementario sección S5): edad (calculada como la diferencia en días entre fecha de entrevista y la fecha declarada de nacimiento, dividida entre 30,4375 para obtener meses aproximados en formato de punto flotante, con una precisión aproximada de 16 dígitos decimales), sexo, ámbito geográfico (reagrupado en Lima Metropolitana, urbano y rural), tiempo (día del año, día de semana, trimestre y año de encuesta) y pobreza distrital (proporción de hogares en el distrito de residencia con ingresos por debajo de la línea de pobreza, según las proyecciones de los mapas de pobreza del INEI para 2009, 2013 y 2018, previas al año de encuesta). Estas covariables descriptivas son referidas previamente en la literatura ^21^^-^^25^. No se incluyeron variables como nivel educativo ni macrorregión, debido a la codificación heterogénea entre las encuestas.

### Análisis de datos

Los datos fueron registrados en fichas impresas durante la entrevista y posteriormente digitalizados en una base de datos con archivos anonimizados. Para este artículo, los archivos analíticos fueron consolidados homogeneizando los nombres y tipos de variables, y seleccionando a los sujetos que cumplían los criterios de inclusión.

Para cada sujeto se calcularon la ingesta total de energía y la contribución energética de cada categoría Nova usando la tabla compilada de composición de alimentos. Esta tabla incluía datos obtenidos mediante análisis químicos directos realizados de forma continua por el CENAN ^32^, así como información proveniente de fuentes externas ^33^, y de etiquetas de productos recolectados durante las entrevistas.

La contribución energética de los AUP fue la principal variable de resultado del estudio, definida individualmente como el porcentaje de la energía consumida (kcal/24h) que proviene de AUP (variable E4RZ), medido en un día aleatorio encuestado. Al examinar la distribución de esta variable (material suplementario sección S2) se identificó una bimodalidad bajo transformación logarítmica exploratoria, con un pico de no consumidores (E4RZ=0) y otro de consumidores (E4RZ>0). Por esta razón, se buscó una transformación normalizante (Box-Cox), que resultó cercana a la potencia 0,5, es decir a la raíz cuadrada.

En consecuencia, se utilizaron tres indicadores per cápita. Para los resultados descriptivos, se empleó el indicador (1) MAAT, media aritmética de los aportes individuales E4RZ (sin transformar) en la población total de niños. Para los resultados analíticos se utilizaron otros dos indicadores: (2) POCT, porcentaje de individuos que consumieron AUP en la población total de niños, y (3) MRAT, media retransformada, calculada como el cuadrado de la media aritmética de las raíces cuadradas de los aportes E4RZ, pero solo entre los consumidores de AUP. MRAT está expresado en la misma escala porcentual que MAAT y se calcula en un subconjunto de la población total.

MAAT, como «contribución energética» es el indicador habitualmente reportado en la literatura ^22^^-^^25^. La asociación con las covariables fue evaluada mediante dos modelos lineales generalizados: (1) binomial para POCT, y (2) normal para MRAT. POCT y MRAT no son indicadores alternativos para un mismo resultado, sino un conjunto complementario de indicadores y modelos que, en conjunto, expresan la contribución energética de los AUP. Más que dos modelos separados, puede considerarse como un único modelo con dos componentes.

El procesamiento y análisis se realizaron con el software R versión 4.4.2, utilizando los paquetes survey, ggplot y dplyr. Todos los estimados, modelos y límites de confianza fueron ajustados según el diseño y la ponderación muestral (material suplementario secciones S3 a S6). La prepublicación de este articulo está disponible en: https://www.medrxiv.org/content/10.1101/2024.05.28.24308069v1. Los datos y el programa de análisis pueden consultarse en: https://github.com/vipermcs/pdata/blob/main/UPJPEDR2023.zip. Se adjunta a esta publicación un suplemento con detalles técnicos y resultados ampliados.

## RESULTADOS

La tabla 1 presenta la distribución ponderada de las covariables sociales y demográficas correspondiente a los 2887 niños incluidos de ambas encuestas. Se observa que la edad y el sexo se distribuyen de manera uniforme. Por diseño, MONIN sobremuestrea el ámbito urbano y el cuarto trimestre del año, mientras que VIANEV sobremuestrea el ámbito rural y el segundo trimestre, y submuestrea el primero. Todos los resultados presentados son ponderados, ajustando estas diferencias muestrales.

**Tabla 1:** Distribución de niveles de covariables, totales y según encuesta.

Covariable/categoría		Total, n=2887	MONIN, n=723	VIANEV, n=2164
Ámbito				
	Lima Metropolitana	29,2 (24,2 a 34,2)	30,9 (22,8 a 38,9)	26,6 (23,8 a 29,5)
	Urbano	53,1 (48,4 a 57,8)	51,3 (45,1 a 57,4)	55,8 (48,5 a 63,1)
	Rural	17,8 (15,2 a 20,3)	17,9 (14,1 a 21,6)	17,6 (14,7 a 20,5)
Sexo				
	Femenino	49,2 (44,6 a 53,8)	49,8 (42,9 a 56,6)	48,3 (43,2 a 53,3)
	Masculino	50,8 (46,3 a 55,3)	50,2 (43,5 a 57,0)	51,7 (46,8 a 56,7)
Edad				
	6-11 meses	21,5 (18,0 a 25,1)	22,7 (17 a 28,4)	19,8 (17,2 a 22,3)
	12-17 meses	18,7 (16,1 a 21,2)	18,2 (14,3 a 22,1)	19,4 (17,1 a 21,7)
	18-23 meses	19,3 (16,8 a 21,8)	18,3 (14,5 a 22,1)	20,7 (18,2 a 23,2)
	24-29 meses	19,5 (16,8 a 22,2)	19,5 (15,4 a 23,5)	19,7 (16,7 a 22,6)
	30-35 meses	21,0 (18,2 a 23,8)	21,4 (17,1 a 25,7)	20,5 (17,4 a 23,5)
Pobreza				
	0-14%	24,4 (19,9 a 29)	18,4 (12,2 a 24,5)	33,6 (27,1 a 40,2)
	15-19%	14,8 (9,9 a 19,7)	13,3 (5,8 a 20,7)	17,2 (12,2 a 22,2)
	20-39%	35,4 (28,6 a 42,1)	38,9 (28,7 a 49,2)	29,9 (23,1 a 36,7)
	40-90%	25,4 (20,9 a 29,9)	29,4 (23,3 a 35,6)	19,3 (12,9 a 25,7)
Trimestre				
	Enero-marzo	17,7 (13 a 22,5)	25,3 (18,1 a 32,4)	6,3 (1,1 a 11,5)
	Abril-junio	28,2 (21,6 a 34,9)	28,2 (17,5 a 38,9)	28,2 (24 a 32,5)
	Julio-setiembre	22,8 (19,5 a 26,1)	17,1 (12,2 a 22,0)	31,4 (27,5 a 35,3)
	Octubre-diciembre	31,3 (25,2 a 37,3)	29,4 (20,8 a 37,9)	34,1 (26,3 a 41,9)

Estimaciones de porcentajes (intervalos de 95% de confianza), ajustadas al diseño y ponderación de la muestra.

MONIN: Monitoreo Nacional de Indicadores Nutricionales. VIANEV: Vigilancia Alimentaria y Nutricional por Etapas de Vida.

La figura 1 muestra la contribución energética de cada categoría Nova (lado izquierdo, Nova 4 corresponde al indicador MAAT) y el porcentaje de consumidores AUP (lado derecho, corresponde al indicador POCT), tanto en la población total como según categorías de covariables. La tabla 2 presenta las estimaciones detalladas de estas cantidades. El indicador MAAT nacional fue de 27% (IC 95%: 25 a 29). El indicador POCT nacional fue de 86% (IC 95%: 84 a 89). Se observan algunas posibles diferencias según edad, pobreza y ámbito geográfico, las cuales se analizan a continuación mediante modelos ajustados.

**Figura 1:** Distribución (no ponderada) de la muestra según encuestas y covariables.

La figura 1 tiene dos partes. El cuerpo de la parte izquierda presenta las contribuciones de energía según procesamiento para la población total y para la población en cada categoría de cada covariable. El cuerpo de la parte derecha presenta las proporciones de niños que consumen alimentos ultraprocesados (AUP) para el total y cada categoría de cada covariable. En la parte izquierda hay barras apiladas representando en el eje horizontal y con colores diferentes la contribución de energía de cada categoría Nova (1: alimentos no o mínimamente procesados, 2: ingredientes culinarios procesados, 3: alimentos procesados, y 4: alimentos ultraprocesados AUP) que es el indicador MAAT (media aritmética de los aportes individuales [sin transformar] en la población total de niños]. Para AUP se presentan también barras abarcando los intervalos de 95% de confianza. En la parte derecha hay barras de error de color rojo representando en el eje horizontal los intervalos de 95% de confianza del indicador POCT (porcentaje de individuos que consumieron AUP en la población total de niños]. Las escalas horizontales para MAAT y POCT están en la parte superior. La muestra total fue de 2887 niños.

**Tabla 2:** Indicadores de procesamiento según nivel de covariables, totales y según encuesta.

Encuesta	Covariables	N	Contribución Nova 1 %	Contribución Nova 2 %	Contribución Nova 3 %	Contribución Nova 4 (AUP) MAAT (%)	Consumidores de AUP POCT (%)
Total		2887	53 (52 a 55)	13 (13 a 14)	6 (6 a 7)	27 (25 a 29)	86 (84 a 89)
	Sexo masculino	1457	53 (52 a 55)	13 (12 a 14)	7 (6 a 8)	27 (25 a 29)	86 (83 a 90)
	Sexo femenino	1430	53 (51 a 55)	14 (13 a 15)	6 (5 a 7)	27 (25 a 29)	87 (83 a 90)
	Edad 6-11 meses	591	53 (48 a 58)	13 (11 a 15)	4 (3 a 5)	30 (24 a 36)	77 (72 a 83)
	Edad 12-23 meses	1168	54 (52 a 56)	14 (12 a 15)	7 (6 a 8)	25 (23 a 27)	89 (85 a 92)
	Edad 24-35 meses	1128	53 (51 a 55)	14 (12 a 15)	7 (6 a 8)	27 (25 a 29)	89 (85 a 93)
	Ámbito Lima Metropolitana	749	51 (47 a 54)	11 (10 a 13)	6 (5 a 7)	32 (28 a 37)	91 (87 a 95)
	Ámbito urbano	1176	52 (50 a 53)	14 (13 a 15)	7 (6 a 8)	27 (26 a 29)	90 (88 a 92)
	Ámbito rural	962	63 (59 a 66)	16 (13 a 18)	5 (4 a 7)	16 (13 a 20)	68 (59 a 77)
	Periodo 2008	68	49 (45 a 54)	13 (10 a 16)	11 (6 a 16)	27 (22 a 32)	87 (80 a 95)
	Periodo 2009	483	51 (48 a 55)	14 (12 a 16)	7 (6 a 8)	28 (24 a 32)	83 (78 a 88)
	Periodo 2010	172	55 (51 a 59)	16 (13 a 18)	6 (5 a 8)	23 (19 a 27)	83 (74 a 91)
	Periodo 2015	558	53 (51 a 56)	12 (11 a 13)	6 (4 a 7)	29 (26 a 33)	92 (89 a 95)
	Periodo 2016	643	53 (51 a 56)	13 (12 a 14)	6 (5 a 7)	28 (25 a 30)	91 (88 a 94)
	Periodo 2019	963	59 (56 a 61)	11 (10 a 12)	5 (5 a 6)	25 (23 a 28)	88 (86 a 91)
	Pobreza 20%+	1700	54 (52 a 56)	15 (13 a 16)	7 (6 a 8)	24 (21 a 27)	82 (79 a 86)
	Pobreza <20%	1187	52 (50 a 54)	11 (11 a 12)	6 (5 a 6)	31 (29 a 33)	92 (90 a 95)
MONIN		723	52 (50 a 54)	14 (13 a 16)	7 (6 a 8)	27 (24 a 29)	83 (80 a 87)
	Sexo masculino	352	52 (49 a 55)	14 (12 a 15)	8 (7 a 9)	27 (23 a 30)	83 (78 a 89)
	Sexo femenino	371	52 (49 a 55)	15 (13 a 17)	6 (5 a 7)	27 (23 a 30)	84 (79 a 89)
	Edad 6-11 meses	152	50 (43 a 57)	14 (11 a 17)	4 (3 a 6)	31 (23 a 40)	76 (68 a 84)
	Edad 12-23 meses	281	52 (48 a 55)	15 (13 a 17)	8 (7 a 10)	25 (22 a 28)	86 (80 a 92)
	Edad 24-35 meses	290	53 (50 a 56)	14 (12 a 16)	7 (6 a 8)	25 (22 a 28)	86 (80 a 92)
	Ámbito Lima Metropolitana	141	49 (44 a 54)	12 (9 a 14)	6 (5 a 8)	33 (26 a 40)	89 (83 a 96)
	Ámbito urbano	438	50 (48 a 52)	15 (13 a 17)	8 (6 a 10)	27 (24 a 29)	88 (84 a 92)
	Ámbito rural	144	63 (57 a 68)	17 (14 a 20)	5 (3 a 7)	15 (10 a 20)	60 (46 a 75)
	Periodo 2008	68	49 (45 a 54)	13 (10 a 16)	11 (6 a 16)	27 (22 a 32)	87 (80 a 95)
	Periodo 2009	483	51 (48 a 55)	14 (12 a 16)	7 (6 a 8)	28 (24 a 32)	83 (78 a 88)
	Periodo 2010	172	55 (51 a 59)	16 (13 a 18)	6 (5 a 8)	23 (19 a 27)	83 (74 a 91)
	Pobreza 20%+	525	53 (50 a 56)	16 (14 a 18)	7 (6 a 9)	24 (20 a 28)	80 (75 a 86)
	Pobreza <20%	198	51 (48 a 54)	12 (10 a 13)	6 (5 a 7)	31 (28 a 35)	90 (86 a 95)
VIANEV		2164	55 (54 a 56)	12 (11 a 12)	6 (5 a 6)	27 (26 a 29)	91 (89 a 93)
	Sexo masculino	1105	55 (53 a 57)	12 (11 a 13)	6 (5 a 6)	27 (25 a 29)	90 (88 a 93)
	Sexo femenino	1059	55 (53 a 57)	12 (11 a 13)	6 (5 a 7)	28 (25 a 30)	91 (88 a 94)
	Edad 06-11 meses	439	58 (54 a 62)	11 (9 a 13)	4 (2 a 5)	28 (24 a 32)	79 (74 a 84)
	Edad 12-23 meses	887	57 (55 a 59)	12 (11 a 13)	6 (5 a 6)	26 (24 a 28)	93 (90 a 95)
	Edad 24-35 meses	838	52 (50 a 54)	12 (11 a 13)	7 (6 a 7)	29 (27 a 31)	95 (93 a 97)
	Ámbito Lima Metropolitana	608	53 (51 a 55)	11 (9 a 12)	5 (4 a 6)	31 (28 a 33)	94 (92 a 96)
	Ámbito urbano	738	53 (52 a 55)	12 (11 a 13)	6 (5 a 7)	29 (26 a 32)	93 (90 a 95)
	Ámbito rural	818	63 (60 a 66)	13 (12 a 15)	6 (5 a 7)	18 (16 a 21)	79 (75 a 84)
	Periodo 2015	558	53 (51 a 56)	12 (11 a 13)	6 (4 a 7)	29 (26 a 33)	92 (89 a 95)
	Periodo 2016	643	53 (51 a 56)	13 (12 a 14)	6 (5 a 7)	28 (25 a 30)	91 (88 a 94)
	Periodo 2019	963	59 (56 a 61)	11 (10 a 12)	5 (5 a 6)	25 (23 a 28)	88 (86 a 91)
	Pobreza 20%+	1175	57 (55 a 59)	13 (12 a 14)	6 (5 a 7)	24 (22 a 26)	87 (84 a 90)
	Pobreza <20%	989	53 (51 a 55)	11 (10 a 12)	5 (4 a 6)	31 (28 a 33)	94 (92 a 96)

Estimaciones (intervalos de 95% de confianza) ajustadas al diseño y ponderación de la muestra.

Solamente Nova 3 Edad 6-11m y periodo 2008 tuvieron coeficiente de variación>15 (15,4 y 24,5, respectivamente).

AUP: alimentos ultraprocesados (Nova 4). MAAT: media aritmética de los aportes individuales (sin transformar) en la población total. POCT: porcentaje de individuos que consumieron AUP en la población total. MONIN: Monitoreo Nacional de Indicadores Nutricionales. VIANEV: Vigilancia Alimentaria y Nutricional por Etapas de Vida.

La tabla 3 presenta el indicador MAAT desagregado según categoría Nova y grupo de alimentos. Entre los AUP, el grupo de leche y derivados fue el principal contribuyente con 18,6% (IC 95%: 17,2 a 20,0), seguido del grupo de cereales y derivados con 4,5% (IC 95%: 3,6 a 5,5) (material suplementario sección S7).

**Tabla 3:** Contribución energética promedio de cada grupo de alimentos según nivel de procesamiento, totales y según encuesta.

Grupo de alimentos		Total %	MONIN %	VIANEV %
No procesados o mínimamente procesados				
	Cereales y derivados	20,7 (19,6 a 21,8)	20,4 (18,5 a 22,2)	21,1 (20,2 a 22,0)
	Verduras, hortalizas y derivados	0,9 (0,8 a 1,0)	0,9 (0,7 a 1,0)	0,9 (0,8 a 1,0)
	Frutas y derivados	8,6 (7,9 a 9,2)	8,2 (7,2 a 9,2)	9,1 (8,4 a 9,8)
	Pescados y mariscos	0,9 (0,7 a 1,1)	0,6 (0,4 a 0,9)	1,2 (0,9 a 1,6)
	Carnes y derivados	4,9 (4,4 a 5,3)	4,5 (3,8 a 5,2)	5,3 (4,9 a 5,7)
	Leches y derivados	1,9 (1,4 a 2,4)	2,6 (1,8 a 3,5)	1,0 (0,6 a 1,3)
	Huevos y derivados	3,2 (2,9 a 3,4)	2,8 (2,4 a 3,2)	3,7 (3,4 a 3,9)
	Leguminosas y derivados	1,8 (1,5 a 2,2)	1,9 (1,3 a 2,5)	1,8 (1,5 a 2,1)
	Tubérculos, raíces y derivados	5,6 (5,1 a 6,1)	4,7 (4 a 5,5)	6,7 (6 a 7,3)
	Otros	2,3 (1,7 a 2,8)	3,2 (2,3 a 4,2)	1,1 (0,7 a 1,4)
Ingredientes				
	Grasas, aceites y oleaginosas	6,2 (5,0 a 7,4)	8 (5,8 a 10,1)	3,9 (3,4 a 4,4)
	Productos azucarados	7,5 (6,8 a 8,2)	7,1 (6 a 8,3)	8,0 (7,3 a 8,7)
	Otros	0,3 (0,2 a 0,4)	0,2 (0,1 a 0,4)	0,4 (0,2 a 0,7)
Procesados				
	Cereales y derivados	4,9 (4,5 a 5,3)	5,1 (4,5 a 5,7)	4,6 (4,1 a 5,1)
	Leches y derivados	0,6 (0,5 a 0,7)	0,5 (0,3 a 0,7)	0,7 (0,6 a 0,9)
	No es un alimento simple	0,8 (0,3 a 1,3)	1,4 (0,6 a 2,3)	0,0 (0,0 a 0,0)
	Otros	0,2 (0,1 a 0,3)	0,2 (0,1 a 0,2)	0,3 (0,1 a 0,4)
Ultraprocesados (AUP)				
	Cereales y derivados	4,5 (3,6 a 5,5)	4,7 (3,0 a 6,3)	4,3 (3,9 a 4,8)
	Leches y derivados	18,6 (17,2 a 20)	17,7 (15,4 a 20,0)	19,8 (18,3 a 20,0)
	Productos azucarados	1,1 (0,7 a 1,4)	1,2 (0,6 a 1,8)	0,9 (0,7 a 1,2)
	Otros	4,6 (4,0 a 5,1)	4,1 (3,1 a 5,0)	5,2 (4,6 a 5,8)

Estimaciones MAAT (intervalos de 95% de confianza) ajustadas al diseño y ponderación de la muestra.

MAAT: media aritmética de los aportes individuales (sin transformar) en la población total. MONIN: Monitoreo Nacional de Indicadores Nutricionales. VIANEV: Vigilancia Alimentaria y Nutricional por Etapas de Vida.

La tabla 4 y la figura 2 presentan los resultados de los modelos ajustados. En el modelo binomial, el indicador POCT aumenta claramente con la edad y disminuye con la pobreza. En el modelo normal, el indicador MRAT disminuye con la edad, aunque muestra un leve incremento en los grupos de mayor edad, y es algo menor en el verano austral y mayor en el otoño. Las variaciones según edad no siguen una tendencia lineal. El modelamiento no identificó asociaciones significativas con otras covariables (sexo, ámbito geográfico, año de encuesta, día del año o del mes), ni con términos de interacción. La revisión de supuestos y diagnósticos de los modelos (material suplementario sección S6) indica que las distribuciones de residuos son satisfactorias, con algunas significancias atribuibles al error tipo I.

**Tabla 4:** Modelos de Indicadores de consumo de alimentos ultraprocesados según niveles de covariables, totales y según encuesta.

Encuesta	Modelo	Término	Pendiente	Error estándar	Valor de p
Total	Binomial	Intercepto	0,377	0,650	0,562
		Edad, logaritmo	0,943	0,207	0,000
		Pobreza distrital, %	-0,036	0,005	0,000
	Normal	Intercepto	10,917	1,876	0,000
		Edad, m	0,145	0,046	0,002
		Edad, logaritmo	-3,088	0,945	0,001
		Abril-Junio	0,614	0,244	0,012
		Julio-Setiembre	0,296	0,204	0,148
MONIN	Binomial	Octubre-Diciembre	0,284	0,214	0,186
		Intercepto	0,680	0,876	0,438
		Edad, logaritmo	0,800	0,276	0,004
		Pobreza distrital, %	-0,037	0,007	0,000
	Normal	Intercepto	11,248	2,527	0,000
		Edad, m	0,133	0,064	0,039
		Edad, logaritmo	-3,124	1,282	0,016
		Abril-junio	0,878	0,322	0,007
		Julio-setiembre	0,452	0,284	0,113
		Octubre-diciembre	0,218	0,250	0,384
VIANEV	Binomial	Intercepto	-0,615	0,561	0,273
		Edad, logaritmo	1,316	0,195	0,000
		Pobreza distrital, %	-0,029	0,006	0,000
	Normal	Intercepto	9,655	1,578	0,000
		Edad, m	0,142	0,040	0,001
		Edad, logaritmo	-2,612	0,806	0,001
		Abril-junio	0,154	0,434	0,723
		Julio-setiembre	0,113	0,431	0,794
		Octubre-diciembre	0,289	0,483	0,551

Estimaciones ajustadas al diseño y ponderación de la muestra. La ponderación de ambas encuestas se ha homologado reescalándola a la población proyectada para cada año.

Modelo normal de MRAT: media retransformada (cuadrado de la media aritmética de las raíces cuadradas de los aportes individuales solamente en la población de consumidores de alimentos ultraprocesados). Modelo binomial de POCT: porcentaje de individuos que consumieron alimentos ultraprocesados en la población total. MONIN: Monitoreo Nacional de Indicadores Nutricionales. VIANEV: Vigilancia Alimentaria y Nutricional por Etapas de Vida.

**Figura 2:** Modelamiento de indicadores según covariables.

La figura 2 presenta los modelos ajustados a las covariables en cuatro paneles. El modelo binomial (n=2887) está en los dos paneles inferiores. El modelo normal (n=2531) está en los dos paneles superiores. Cada panel presenta el escenario para una de las covariables en cada modelo. El eje vertical presenta la variable dependiente. Para el modelo binomial la dependiente es el indicador POCT (porcentaje de individuos que consumieron alimentos ultraprocesados [AUP] en la población total de niños). Para el modelo normal la dependiente es el indicador MRAT (media retransformada, cuadrado de la media aritmética de las raíces cuadradas de los aportes individuales solamente en la población de niños consumidores de AUP). El eje horizontal presenta la covariable. Las líneas sólidas azules presentan la predicción para un niño «promedio» (21 meses de edad, en julio-septiembre, 30% de pobreza distrital, excepto por la covariable de cada panel). Las sombras celestes son las bandas de 95% de confianza. Los círculos negros son los estimados ponderados de la muestra observada (con su mezcla real de covariables) y las barras grises son sus intervalos ponderados de 95% de confianza. Estos modelos finales no seleccionaron términos de interacción.

## DISCUSIÓN

Este estudio constituye la primera estimación del consumo de AUP en niños del Perú. El consumo se describió mediante dos indicadores: MAAT, la contribución energética promedio de AUP fue de 27% (IC 95%: 25-29), y POCT, el porcentaje de consumidores de AUP fue de 86% (IC 95%: 84-89). Asimismo, el análisis que incorporó un tercer indicador (MRAT, la contribución energética promedio solo entre consumidores) identificó como covariables asociadas la edad (POCT aumenta con la edad, mientras que MRAT disminuye), la pobreza (MRAT es menor a mayor pobreza) y el trimestre calendario (MRAT es menor en verano austral y mayor en otoño).

Cuando se compara la contribución energética de los AUP en el Perú (27% en niños entre 6 y 35 meses), con la del grupo etario más cercano disponible para otros países (niños de 2 a 5 años) ^21^, se observa que el valor peruano es menor que el reportado en México (38%), Chile (44%), Australia (47%), Estados Unidos (58%), Reino Unido (61%), pero mayor que el registrado en Colombia (18%). Tomando en cuenta el modelamiento, y pese a posibles diferencias metodológicas menores, se postula que, cuando en un futuro se calcule para niños de 2 a 5 años, la cifra comparable para Perú sea algo menor, pero en la misma posición entre países.

Se observó que la mayor parte del consumo de AUP proviene de alimentos pertenecientes a dos grupos: leche y derivados, y cereales. La leche evaporada es el AUP más frecuente, consumido por 58% (IC 95%: 55 a 61) de los niños, y aportó el 13% (IC 95%: 11 a 14) de la energía total, aproximadamente la mitad de la energía atribuida a los AUP. En contraste, la leche fresca fue consumida solo por el 7% (IC 95%: 6 a 9). La leche evaporada contiene sodio, grasas saturadas y aditivos ^32^^,^^33^, y promueve el consumo de azúcares añadidos cuando se endulza en el hogar (los niños que la consumieron ingirieron un promedio geométrico de 10 g (IC 95%: 9 a 12) de azúcar, frente a 4 g (IC 95%: 4 a 5) consumidos por quienes no la consumieron. Por otro lado, proporciona varios macro y micronutrientes, constituye una fuente de fortificación y no requiere refrigeración mientras la lata permanezca cerrada. La leche evaporada representa una industria importante en Perú desde aproximadamente 1940 ^35^. El balance entre estas consideraciones en favor o en contra es un tema que requiere mayor investigación, con el fin de generar evidencia que permita revisar las recomendaciones sobre su uso en la alimentación infantil.

Se escogió un conjunto limitado de covariables, correspondientes a las utilizadas en estudios previos ^22^^-^^25^, con excepción del dominio macrorregional, cuyas categorías son específicas de cada país, y escolaridad de la madre, que en Perú es muy homogénea.

Respecto de la edad, en otros países latinoamericanos, con niños mayores de 2 años, MAAT, la contribución energética, disminuye con la edad ^22^^,^^24^^,^^25^. El presente estudio evidenció algo más complejo: MRAT, la contribución energética promedio, de AUP solo en consumidores, disminuyó con la edad hasta aproximadamente 21 meses y, a partir de ese punto, pareció aumentar ligeramente, mientras que POCT, el porcentaje de consumidores AUP, se incrementó con la edad. Se interpreta que en MAAT el componente opuesto de POCT (es decir, los sujetos que no consumieron AUP) queda enmascarado. Una posible explicación sería la introducción de alimentos para lactantes, como los del grupo leche y derivados (particularmente la leche evaporada) y los cereales. La contribución energética de estos alimentos se reduciría posteriormente, cuando se introduzca la dieta del hogar. La muestra utilizada no permite modelar con precisión el tercer año de vida, y la literatura tampoco ofrece datos para los primeros dos años.

Respecto del nivel socioeconómico se ha reportado también alguna evidencia ^(^^22^^,^^25^^)^ de diferencias similares a las observadas en el presente análisis, el cual encontró que MRAT decrece con el nivel de pobreza. Asimismo, existe evidencia de un grado variable de asociación ^(^^36^, aunque la mayoría de los estudios son en adultos que participaron en la National Health and Nutrition Examination Survey (NHANES) ^37^^-^^39^. En un contexto en el que las ventas de AUP están en aumento ^14^, comprender los mecanismos que relacionan AUP, pobreza y precios resulta de importancia para diseñar intervenciones eficaces ^40^.

Respecto de las diferencias trimestrales, aunque plausibles, no se encontraron reportes en la literatura revisada. La estacionalidad podría estar relacionada con la disponibilidad de AUP, el clima, los ingresos y/o los gastos; sin embargo, estas hipótesis no han sido documentadas previamente y merecen ser investigadas en estudios futuros. Tampoco se identificaron diferencias según el sexo. De igual manera, no se hallaron diferencias por año, lo cual podría explicarse si el nivel de pobreza, que se ha reducido marcadamente en Perú durante el período observado ^41^, fuese el principal mediador de los cambios a lo largo del tiempo.

Los hallazgos presentados tienen implicancias relevantes para la salud pública y nutrición. En primer lugar, aunque no hay límites oficiales internacionales para calificar la importancia sanitaria de los niveles de consumo de AUP, dado que no son alimentos esenciales y considerando los riesgos mencionados, resulta razonable mantener esos niveles al mínimo posible. En segundo lugar, si existiesen AUP individuales con beneficios, tendrían que discutirse y justificarse, en ese sentido la leche evaporada constituye una prioridad para estudio, como se ha discutido previamente. En tercer lugar, el conjunto de tres indicadores utilizados en este estudio, además de otros como los nutrientes críticos ^16^, podrían incorporarse a los instrumentos de vigilancia, ya que aportan información no redundante y contribuye a afinar la descripción y el análisis. En cuarto lugar, las normas peruanas de etiquetado de alimentos ^(^^42^ exigen símbolos octogonales de advertencia en el empaquetado, una intervención que debe continuar y reforzarse considerando también a la población infantil, y cuyo monitoreo y evaluación requiere vigilar las tendencias de AUP durante los próximos años. En quinto lugar, aunque las covariables identificadas aquí son descriptivas, pueden servir de base para el diseño de estudios orientados a focalizar grupos o mecanismos de intervención.

Entre las limitaciones de este estudio se identifican las siguientes: (1) representatividad de áreas rurales (aunque formalmente incluidas con la ponderación adecuada, se tiene la impresión de que las dificultades de campo pueden haber generado pérdidas no muy bien documentadas); (2) pérdida al recordatorio (siendo un procedimiento largo que requiere citas para los días aleatorios, es frecuente en encuestas de recordatorio ^(^^43^^,^^44^ que no se logren concertar o llevar a cabo; la disposición de reemplazos preserva el tamaño muestral meta, pero no necesariamente compensa los posibles sesgos; a manera de un estimado grueso, de los niños elegibles en el archivo consolidados, 96% han sido incluidos, de los niños aparentemente elegibles en los archivos de origen, 70% han sido seleccionados en el archivo consolidado); (3) reproducibilidad de la clasificación Nova, especialmente para los AUP (como se ha reportado en algunos trabajos previos ^45^^-^^47^) y es también la experiencia del equipo, puede haber cierta ambigüedad en la asignación de la categoría de AUP, particularmente cuando la descripción de los alimentos puede incluir versiones con y sin AUP. Si bien se considera que estos casos no son numerosos ni abarcan la mayoría de la masa consumida, pero se puede descartar esta eventualidad); (4) la selección de las covariables, cuya intención ha sido básicamente descriptiva (entre otras variables que pudieran ser de interés, pero que no han sido incluidas aquí podemos mencionar las características de la educación materna, la región ecológica y los precios y, elasticidades de los alimentos); (5) la medición de la covariable pobreza, que es una proyección distrital, no una calificación individual, lo cual podría atenuar su efecto; y (6) la medición del consumo, que es con un solo recordatorio, no estima el consumo usual, por lo cual la variabilidad observada es algo mayor. Si bien algunas de estas limitaciones pueden haber producido sobreestimaciones y otras, subestimaciones, no debilitan las conclusiones del estudio. No obstante, todos estos aspectos merecen ser abordados en futuras investigaciones.

Entre las fortalezas de nuestro estudio destacan: (1) cobertura de toda la diversidad regional; (2) tamaño muestral grande, como se refleja en los intervalos de confianza; (3) aplicación de un R24h estandarizado; (4) aplicación del R24h en días aleatorios; y (5) aplicación de ajustes estadísticos a la bimodalidad (inflación de ceros), asimetría, confusión y muestra compleja. Estos resultados pueden servir como línea de base para el análisis de la ingesta de AUP en niños del Perú antes de la implementación del etiquetado frontal.

Se concluye que, entre los niños de 6 a 35 meses de edad en Perú entre el 2008 y 2019, los AUP (Nova 4): (1) contribuyen con el 27% de la energía total; (2) fueron consumidos por el 86% de los niños, un porcentaje que aumentó con la edad y disminuyó con la pobreza; (3) los AUP con mayor aporte de energía son los del grupo de leche y derivados, y cereales; y (4) entre los consumidores, la contribución energética disminuyó con la edad y presentó variación estacional. Se recomienda que, dada la creciente evidencia contra los AUP, la tendencia temporal debería ser vigilada también en este grupo de edad. Debe revisarse la conveniencia o no de desmotivar el consumo de leche evaporada y cereales en este grupo. Finalmente, resulta pertinente un análisis más profundo del patrón de consumo de los AUP en niños de 6 a 59 meses, periodo caracterizado por una dinámica nutricional particularmente importante.
